# Isoliquiritigenin alleviates early brain injury after experimental intracerebral hemorrhage via suppressing ROS- and/or NF-κB-mediated NLRP3 inflammasome activation by promoting Nrf2 antioxidant pathway

**DOI:** 10.1186/s12974-017-0895-5

**Published:** 2017-06-13

**Authors:** Jun Zeng, Yizhao Chen, Rui Ding, Liang Feng, Zhenghao Fu, Shuo Yang, Xinqing Deng, Zhichong Xie, Shizhong Zheng

**Affiliations:** 1 Department of Neurosurgery, Zhujiang Hospital, The National Key Clinical Specialty, The Neurosurgery Institute of Guangdong Province, Guangdong Provincial Key Laboratory on Brain Function Repair and Regeneration, The Engineering Technology Research Center of Education Ministry of China, Southern Medical University, Guangzhou, 510282 China; 2 Department of Neurosurgery, Jingmen No. 1 People’s Hospital, Jingmen, 448000 Hubei China; 3 Department of Neurosurgery, Affiliated Hospital of Xiangnan University, Chenzhou, 423000 Hunan China; 4 Department of Neurosurgery, The Fifth Affiliated Hospital of Southern Medical University, Guangzhou, 510900 Guangdong China; 5 Department of Neurosurgery, Gaoqing Campus of Central Hospital of Zibo, Gaoqing People’s Hospital, Gaoqing, Zibo, 256300 Shandong China; 6 Department of Neurosurgery, 999 Brain Hospital, Jinan University, Guangzhou, 510510 Guangdong China

**Keywords:** ICH, Early brain injury, ILG, Nrf2, ROS, NF-κB, NLRP3 inflammasome

## Abstract

**Background:**

Intracerebral hemorrhage (ICH) induces potently oxidative stress responses and inflammatory processes. Isoliquiritigenin (ILG) is a flavonoid with a chalcone structure and can activate nuclear factor erythroid-2 related factor 2 (Nrf2)-mediated antioxidant system, negatively regulate nuclear factor-κB (NF-κB) and nod-like receptor family, pyrin domain-containing 3 (NLRP3) inflammasome pathways, but its role and potential molecular mechanisms in the pathology following ICH remain unclear. The present study aimed to explore the effects of ILG after ICH and underlying mechanisms.

**Methods:**

ICH model was induced by collagenase IV (0.2 U in 1 μl sterile normal saline) in male Sprague-Dawley rats weighing 280–320 g. Different doses of ILG (10, 20, or 40 mg/kg) was administrated intraperitoneally at 30 min, 12 h, 24 h, and 48 h after modeling, respectively. Rats were intracerebroventricularly administrated with control scramble small interfering RNA (siRNA) or Nrf2 siRNA at 24 h before ICH induction, and after 24 h, ICH model was established with or without ILG (20 mg/kg) treatment. All rats were dedicated at 24 or 72 h after ICH. Neurological deficits, histological damages, brain water content (BWC), blood-brain barrier (BBB) disruption, and neuronal degeneration were evaluated; quantitative real-time RT-PCR (qRT-PCR), immunohistochemistry/immunofluorescence, western blot, and enzyme-linked immunosorbent assay (ELISA) were carried out; catalase, superoxide dismutase activities and reactive oxygen species (ROS), and glutathione/oxidized glutathione contents were measured.

**Results:**

ILG (20 and 40 mg/kg) markedly alleviated neurological deficits, histological damages, BBB disruption, brain edema, and neuronal degeneration, but there was no significant difference between two dosages. ILG (20 mg/kg) significantly suppressed the NF-κB and NLRP3 inflammasome pathways and activated Nrf2-mediated antioxidant system. Gene silencing of Nrf2 aggravated the neurological deficits, brain edema, and neuronal degeneration and increased the protein levels of NF-κB p65, NLRP3 inflammasome components, and IL-1β. ILG delivery significantly attenuated the effects of Nrf2 siRNA interference mentioned above.

**Conclusions:**

Intraperitoneal administration of ILG after ICH reduced early brain impairments and neurological deficits, and the mechanisms were involved in the regulation of ROS and/or NF-κB on the activation of NLRP3 inflammasome pathway by the triggering of Nrf2 activity and Nrf2-induced antioxidant system. In addition, our experimental results may make ILG a potential candidate for a novel therapeutical strategy for ICH.

**Electronic supplementary material:**

The online version of this article (doi:10.1186/s12974-017-0895-5) contains supplementary material, which is available to authorized users.

## Background

Spontaneous intracerebral hemorrhage (ICH) belongs to a fatal cerebrovascular disorder, accounting for 15 to 20% in all stroke types, commonly accompanied with high morbidity and mortality [1, 2]. Brain injury after ICH is broadly classified as primary brain injury and secondary brain injury [3, 4]. Primary brain injury occurring within first several hours post ICH is caused by the hemorrhage and growth of hematoma which lead to the mechanical impairments and compression of adjacent cerebrovascular architecture [1, 3–5]. Hematoma size is a powerful and easy-to-use predictor of 30-day mortality and morbidity in patients with ICH, and large hemorrhage often indicates a poor prognosis [4, 6]. Blood components extravasated from the ruptured blood vessels and degradation products of blood cells can induce severe secondary brain injury including neurobehavioral deterioration, brain cell death, cerebral edema, and blood-brain barrier (BBB) disruption [1, 3–5]. Though the understanding of pathophysiological mechanisms to brain injury after ICH has been well improved in recent decades, there are still no effective therapies being available for the prevention of ICH-induced brain impairments [3–5, 7]. Furthermore, increasing evidences have shown that inflammatory response and oxidative stress which occur following ICH play a key role in pathophysiological processes of ICH-induced early brain dysfunctions [3–5, 7, 8].

Nuclear factor erythroid-2 related factor 2 (Nrf2) is a key transcription factor and master regulator of the cellular response of oxidative stress, which can induce the expression of antioxidant and detoxification enzymes and downstream proteins such as NAD(P)H: quinone oxidoreductase-1 (NQO1), catalase (CAT), superoxide dismutase (SOD), heme oxygenase-1 (HO-1), glutathione peroxidase (GPX), and glutathione-S-transferase (GST) [9, 10]. Recent study report showed that the expression of Nrf2 was gradually increased following ICH at 2 h, peaked at 24 h, and then slightly decreased with time until 10 days [11]. In addition, Nrf2 has been identified to hold the neuroprotective effects against the early brain injury after ICH by translocating into nucleus after being activated, binding to the antioxidant response element (ARE), then initiating the expression of a series of antioxidant and detoxification enzymes and proteins, as a result, improving neurological deficits, alleviating brain edema, and decreasing the infiltration of inflammatory cells [11–15].

The NLRP3 (NALP3, cryopyrin) inflammasome [NLR (Nod-like receptor) family, pyrin domain-containing 3 inflammasome], a best characterized member of NLR family and one of the key components of innate immune system, has been reported by others [8, 16] and us [17] to take part in the processes of early brain injury after ICH via facilitating caspase-1 and interleukin-1beta (IL-1β) processing, which amplifies the inflammatory response and blockade or knockdown of NLRP3 inflammasome can alleviate the brain damages [8, 16, 17]. Recently, reports have indicated that Nrf2 could negatively regulate NLRP3 inflammasome activity by inhibiting reactive oxygen species (ROS)-induced NLRP3 inflammasome activation [18, 19]. However, the relationship between Nrf2 antioxidant pathway and NLRP3 inflammasome activation and whether Nrf2 reduces the early brain injury via the suppression of NLRP3 inflammasome and whether the above-mentioned inhibitory effect is involved in Nrf2 mediated ROS and/or nuclear factor-κB (NF-κB) suppression have not been explored in the experimental rat ICH model.

Isoliquiritigenin (ILG), a component of *Glycyrrhiza uralensis* (*G. uralensis*), is a flavonoid with a chalcone structure and it holds multiple biological activities [20]. Recent papers have shown that ILG was a potent inhibitor of NLRP3 inflammasome [21, 22] and NF-κB [23–25], thus exerting a protective effect. Also, there were reports showing that ILG could activate Nrf2-mediated antioxidant pathway via promoting Nrf2 translocation into the nucleus and then initiating a series of genes to express [9, 26–29]. However, it remains unclear whether ILG has a protective effect against the early brain injury following ICH, and the detailed molecular mechanisms have not been elucidated. Thus, in this study, we are attempting to explore the effects of ILG on the early brain injury after an experimental rat intracerebral hemorrhage model and the potential molecular mechanisms.

## Methods

### Animals

Adult male Sprague-Dawley rats (SD rats) weighing between 280 and 320 g (8–10 weeks) were obtained from the Animal Experiment Center of Southern Medical University. All experimental procedures and animal care were approved by the Southern Medical University Ethics Committee and were conducted in accordance with the guidelines of the National Institutes of Health on the care and use of animals. All rats were housed in a light-, temperature-, and humidity-controlled specific pathogen-free (SPF) environment (under a 12-h light/dark cycle with constant temperature about 25 °C and relative humidity approximating 55%). All rats had free access to standard food and water during the experiments.

### Experimental design and groups

Experiments were conducted in a rat model of collagenase type IV-induced ICH. In the first experiment, 180 rats were used (183 rats suffered to the surgery, 180 rats survived) to evaluate the effects of ILG on the early brain injury post ICH. The rats were randomly and evenly assigned to five groups of 36 rats each, namely, sham group, ICH + vehicle-1 [dimethylsulfoxide (DMSO)] group, ICH + ILG 10 mg/kg group, ICH + ILG 20 mg/kg group, and ICH + ILG 40 mg/kg group. All rats in this experiment were evaluated with a Modified Neurological Severity Score (mNSS) (*n* = 12) scale at 24 or 72 h after ICH, except for the rats that perform extravasation detection of Evans blue (EB) dyes (*n = 6*) at the same time points. Then, the rats were killed, and brain tissue samples were taken to perform brain water content (BWC) measurements (*n* = 6), hematoxylin and eosin (H&E) staining (*n* = 6), and Fluoro-Jade® C (FJC) staining (*n* = 6).

In the second experiment, 120 rats were used (122 rats experienced the operation, 120 rats survived) to explore the underlying molecular mechanisms of ILG’s effects on the early brain injury after ICH. The rats were randomized into four groups (30 rats per group): sham group, ICH group, ICH + vehicle-1 (DMSO) group, and ICH + ILG 20 mg/kg group. All rats in the experiment were sacrificed at 24 h after ICH for real-time reverse transcription-quantitative polymerase chain reaction (RT-qPCR) (*n* = 6), western blot (WB) (*n* = 6), immunohistochemistry (IHC)/immunofluorescence (IF) (*n* = 6), enzyme-linked immunosorbent assay (ELISA) (*n* = 6), and glutathione/oxidized glutathione (GSH/GSSG) contents, ROS content, CAT activity, and SOD activity analyses (*n* = 6).

In the third experiment, 132 rats were used (132 of 137 rats after the surgery survived) to execute the study on effects of Nrf2 small interfering RNA (siRNA) interference and Nrf2 siRNA together with ILG co-administration on early brain injury following ICH. The rats were randomly divided into six groups (sham group, ICH + vehicle-2 [mixtures of Entranster^TM^ in vivo transfection reagent and siRNA diluent (RNase-free H_2_O)] group, ICH + control scramble siRNA group, ICH + Nrf2 siRNA group, ICH + Nrf2 siRNA + vehicle-1 group, ICH + Nrf2 siRNA + ILG 20 mg/kg group). All rats were decapitated to perform related RT-qPCR (*n* = 6), WB (*n* = 6), mNSS scoring (*n* = 6), BWC (*n* = 6), FJC staining analyses (*n* = 6).

### ICH model

The procedure for ICH model in rats has been described in previous publications with some small modifications [17, 30]. In brief, the rats were anesthetized by intraperitoneal injection (i.p.) of pentobarbital sodium (45 mg/kg). Then, the animals were placed in a rat brain stereotaxic apparatus and under aseptic condition. Rectal temperature was maintained at 37 °C throughout the surgical procedure using an insulation board connected with water bath circulation system. Next, a midline incision on the scalp to expose the skull and bregma and a cranial burr hole (1 mm in diameter) was drilled in the right part of the brain, a 5-μl microsyringe with a needle tip (Shanghai high pigeon industry & trade co., LTD, Shanghai, China) was inserted stereotactically through the burr hole and into the right striatum which coordinates were 0.1 mm anterior, 3.5 mm lateral, and 6.0 mm ventral to the bregma. Collagenase type IV (0.2 U in 1 μl sterile normal saline) was administrated over a period of 10 min via stereotaxic intrastriatal injection. The needle was kept in situ for an additional 10 min to prevent back-flow. Then, the microsyringe was slowly removed and the craniotomy was sealed with bone wax. Finally, the wound was sutured. The sham-operated rats were treated via the same way except that they were administrated 1 μl sterile normal saline into the right striatum. The rats were allowed to recover in separate cages with free access to food and water.

### In vivo siRNA transfection and drug delivery

The transfection of Nrf2 siRNA for rat brains in vivo were conducted according to the method described formerly [17, 31, 32]. Briefly, the rats were placed under anesthesia, then a cranial burr hole (1 mm in diameter) was drilled, following a 25-μl microsyringe with a needle tip (Shanghai high pigeon industry & trade co., LTD, Shanghai, China) was inserted stereotaxically into the right lateral ventricle. The stereotaxic coordinates were 1.5 mm posterior, 1.0 mm lateral, and 3.2 mm below the horizontal plane of the bregma [32]. Nrf2 siRNA (sc-156128, Santa Cruz biotechnology, USA) and control scramble siRNA (sc-37007, Santa Cruz Biotechnology, USA) were applied with in vivo transfection reagent (Entranster^TM^-in vivo, 18668-11-1, Engreen Biosystem Co, Ltd., Beijing, China) at 24 h before modeling by intracerebroventricular injection [31, 32]. The microsyringe was left in place for an additional 10 min after administration and then slowly withdrawn. At last, the incision was closed with sutures. The sham-operated rats received a cranial burr hole, but only a needle was inserted.

ILG (1811912, Shanghai Macklin Biochemical Co., Ltd., Shanghai, China) was dissolved into DMSO (D5879, Sigma-Aldrich) solution (20 mg/ml). The rats were administrated intraperitoneally with either ILG at 10, 20, and 40 mg/kg or the same volume of DMSO at 30 min, 12 h, 24 h, and 48 h after ICH induction.

### Behavioral assessment

We used a mNSS scale [33, 34] to assess the behavioral deficits at 24 h and 72 h after ICH, which was performed by two trained investigators and both of whom had been blinded to animal grouping. The mNSS is consisted of motor, sensory, balance, and reflex tests. Neurological function is graded via the scale of 0–18 points (1–6, mild injury; 7–12, moderate injury; 13–18, severe injury; the scores of 0 and 18 represent normal performance and severe neurological deficit, respectively). In the severity scores of neurological function injury, 1 score point is obtained for the incapacity to complete the test or the absence of a tested reflex. Thus, a higher score indicates a more severe neurological injury [33–35].

### Measurement of BWC

BWC was evaluated via a wet/dry weight method, as previously described [36, 37]. Briefly, at 24 or 72 h after ICH, the rats were deeply anesthetized with an i.p. of pentobarbital sodium and then were decapitated. The brain of the rats were immediately removed and separated into five parts, namely ipsilateral and contralateral cortex, ipsilateral and contralateral basal ganglia, and cerebellum. The cerebellum was used as an internal control. Each part was placed on a pre-weighed piece of aluminum foil and obtained the wet weight by an electric analytic balance, and then was dried at 100 °C for 24 h in an electric oven to get the dry weight. BWC was assessed using the following formula: [(wet weight − dry weight)]/(wet weight) × 100%.

### Evaluation of BBB permeability

Quantitative analysis of BBB permeability was evaluated via EB dye (Wako Pure Chemical Industries, Ltd., Japan) extravasation, as described previously with minor modifications [38–40]. Briefly, the rats were anesthetized and administrated intravenously 2% EB solution in normal saline (4 ml/kg) by the femoral vein. After a circulation of 2 h, intracardiac perfusion was performed under deep anesthesia with 0.01 M phosphate buffer solution (PBS) (pH 7.4) of 250 ml to clear EB dyes in cerebral circulation. Subsequently, the brains were removed and the brain samples were immediately separated into the left hemisphere and right hemisphere. Tissue samples were then incubated in 50% trichloroacetic acid solution (2 ml). Following homogenization and centrifugation (15,000 rpm for 20 min), the supernatant (1 ml) was diluted with ethanol (1: 3), and its fluorescence intensity was measured at an excitation wavelength of 620 nm and an emission wavelength of 680 nm with an automatic microplate reader. The EB dye leakage was expressed as micrograms per gram brain weight.

### Preparation of paraffin-embedded sections

Paraffin-embedded sections were made as previously described [17, 35, 40] with some modifications. After deep anesthetization with pentobarbital sodium, the rats were transcardially perfused with 250 ml of 0.01 M PBS (pH 7.4) followed by 500 ml 4% paraformaldehyde solution. And then, the brains were removed and post-fixed by immersion in the same fixative solution at 4 °C for 24–48 h. After dehydration and vitrification, tissue samples were embedded in paraffin, and 4-μm sections were prepared. The sections were then dewaxed in xylene, re-hydrated in graded ethanol and deionized water, and then processed for H&E, IHC, IF, and FJC staining.

### H&E staining

The coronal brain sections (4-μm thickness, paraffin-embedded) were prepared as mentioned above, then were stained with eosin for 10 s followed by hematoxylin re-staining for 5 min. After dehydrated in graded ethanol and cleared in xylene, slides were mounted by neutral balsam. Images were obtained using a microscope (Leica-DM2500, Germany).

### IHC staining

IHC staining was conducted as described previously [35, 36] with a few modifications. Coronal paraffin-embedded brain sections (4-μm thickness) were prepared as before-mentioned and antigen retrieval was performed by heat treatment in a microwave oven for 21 min in Tris–ethylene diamine tetraacetic acid (EDTA) buffer solution (0.05 mol/l Tris, 0.001 mol/l EDTA; pH 8.5). Endogenous peroxidase activity was inactivated using 0.3% H_2_O_2_ for 10 min followed by washing with PBS. After blocking by 5% bovine serum albumin (BSA) for 20 min, the slides were incubated overnight at 4 °C with the following primary antibodies used: rabbit monoclonal anti-NF-κB p65 (D14E12) XP® antibody (1:800, #8242, Cell Signaling Technology); rabbit polyclonal anti-Nrf2 (L593) antibody (1:200, BS1258, Bioworld); rabbit polyclonal anti-HO-1 antibody (1:200, BS6626, Bioworld); goat polyclonal anti-NQO1 antibody (1:200, ab2346, Abcam); mouse monoclonal anti-Cryopyrin (NLRP3) (6F12) antibody (1:100, sc-134306, Santa Cruz Biotechnology); mouse monoclonal anti-3-Nitrotyrosine (3-NT) antibody [39B6] (1:200, ab61392, Abcam); mouse monoclonal anti-8-Hydroxyguanosine (8-OHdG) antibody [N45.1] (1:200, ab48508, Abcam); rabbit polyclonal anti-Iba-1 antibody (1:600, WAKO, Osaka, Japan); and mouse monoclonal anti-CD68 antibody [ED1] (1:200, ab31630, Abcam). After washing with PBS, the sections were incubated with biotinylated goat anti-mouse IgG, goat anti-rabbit IgG, and donkey anti-goat IgG secondary antibodies for 20 min and then incubated with horseradish peroxidase (HRP)-streptavidin reagent for 20 min. Finally, immunoreactivity was detected using 3,3-diaminobenzidine (DAB), followed by re-staining with hematoxylin. Images were obtained by using a microscope (Leica-DM2500, Germany). The number of immunopositive cells in the perihematomal region was counted in a blinded manner and was expressed as number/0.1 mm^2^ areas.

### IF staining

IF staining was performed as described previously [17, 35] with a few modifications. Coronal paraffin-embedded 4-μm thickness brain sections were prepared as mentioned above. Antigen retrieval was conducted as IHC staining. After blocking by 5% BSA for 40 min, sections were incubated overnight at 4 °C with the following primary antibody used: rabbit polyclonal anti-myeloperoxidase (MPO) antibody (1:50, ab9535, Abcam). After washing with PBS, sections were then incubated with the secondary antibody: Alexa Fluor 594 goat anti-rabbit IgG (H + L) (1:100, A-11012, Invitrogen) for 1 h at room temperature. Following washing three times with PBS, the sections were re-stained by 4′6-diamidino-2-phenylindole (DAPI) for 10 min. Then, images were obtained with a fluorescence microscope (ZEISS-AXIO Scope. Al, Germany).

### FJC staining

For the detection of degenerating neuron, FJC staining was conducted as described previously with some modifications [41]. Briefly, rat brain sections were prepared as mentioned above, then sections were rinsed with distilled water and immersed into 0.06% potassium permanganate solution for 10 min followed by transferred into a 0.0001% solution of FJC (AG325, Merck millipore) dissolved in 0.1% acetic acid vehicle for 30 min. After washing with distilled water, the slides were put into an oven at 50 °C for 20 min. The dried slides were then cleared in xylene for 5 min and then coverslipped with DPX mountant for histology (06522-100 ml, Sigma). Four high-power images (×400 magnification) were taken around hematoma using a fluorescence microscope (ZEISS-AXIO Scope, Al, Germany) in each slide. FJC staining positive cells were counted on these four areas.

### Real-time RT-qPCR

Quantitative real-time RT-PCR assessment for the messenger RNA (mRNA) levels was conducted via using Prime Script RT-PCR kits (RR047A and RR820A, Takara) according to the manufacturer’s instructions. The mRNA level of β-actin was used as an internal control. The real-time PCR program steps were 95 °C for 30 s, 40 cycles of 95 °C for 3 s, 60 °C for 34 s. The mRNA level of each target gene was normalized to that of β-actin mRNA. Fold-induction was calculated using the 2^−ΔΔCT^ method, as previously described [42, 43]. The specific sequences of primers used were shown as Table 1.

**Table 1:** Primers used in real-time qRT-PCR reactions

Gene	Forward primer (5′-3′)	Reverse primer (5′-3′)
NLRP3	CGGTGACCTTGTGTGTGCTT	TCATGTCCTGAGCCATGGAAG
ASC	GACAGTACCAGGCAGTTCGT	AGTAGGGCTGTGTTTGCCTC
Caspase-1	GAACAAAGAAGGTGGCGCAT	AGACGTGTACGAGTGGGTGT
IL-1beta	CCTATGTCTTGCCCGTGGAG	CACACACTAGCAGGTCGTCA
IL-18	ACCACTTTGGCAGACTTCACT	ACACAGGCGGGTTTCTTTTG
Nqo1	GTTTGCCTGGCTTGCTTTCA	ACAGCCGTGGCAGAACTATC
HO-1	GGTGATGGCCTCCTTGTACC	GTGGGGCATAGACTGGGTTC
Actin	TCAGCAAGCAGGAGTACGATG	GTGTAAAACGCAGCTCAGTAACA

### WB

WB was performed according to our previous study method [17, 35]. We used the following primary antibodies to perform the WB analyses: rabbit monoclonal anti-NF-κB p65 (D14E12) XP® antibody (1:1000, #8242, Cell Signaling Technology); rabbit polyclonal anti-Nrf2 (L593) antibody (1:500, BS1258, Bioworld); mouse monoclonal anti-Cryopyrin (NLRP3) (6F12) antibody (1:1000, sc-134306, Santa Cruz Biotechnology); rabbit polyclonal anti-PYCARD (ASC) antibody (1:500, A1170, Abclonal); goat polyclonal anti-caspase-1 p20 (M-19) antibody(1:1000, sc-1218, Santa Cruz Biotechnology); rabbit polyclonal anti-IL-1β (H-153) antibody (1: 1000, sc-7884, Santa Cruz Biotechnology); and rabbit polyclonal anti-IL-18 (H-173) antibody (1:1000, sc-7954, Santa Cruz Biotechnology), and glyceraldehyde 3-phosphate dehydrogenase (GAPDH) (1:1000, Cell Signaling Technology) was used as an internal reference. Blot bands were quantified via densitometry with ImageJ software (National Institutes of Health, Baltimore, MD, USA), and protein levels were expressed as the ratio of values of the detected protein bands to that of GAPDH bands.

### Cytokine ELISA assay

At 24 h after ICH, the rats were deeply anesthetized. The serum samples from intracardiac puncture blood and the supernatant samples from perihematomal brain tissue homogenate were obtained and stored at −80 °C until use. Measurements of IL-1β and IL-18 levels were conducted by a double-antibody sandwich ELISA Array Kit according to the reagent manufacturer’s instructions. Briefly, prepared tissue supernatant or serum samples were added to monoclonal antibody enzyme well which is pre-coated with rats IL-1β or IL-18 antibodies labeled with biotin and combined with Streptavidin-HRP to form immune complex, then incubated for 1.5 h in a 37 °C condition and washed three times with PBS to remove the uncombined enzyme. After adding the chromogen solution A and B, the samples were detected using an automatic microplate reader at 450 nm.

### Measurements of CAT activity, SOD activity and ROS content, GSH/GSSG contents

CAT assay kit (visible light) (A007-1), total SOD assay kit (Hydroxylamine method) (A001-1), ROS assay kit (E004), and total GSH/GSSG assay kit (A061-1) were all purchased from Nanjing Jiancheng Bioengineering Institute (Nanjing, China) and were used to measure the CAT activity, SOD activity and ROS content, GSH/GSSG contents according to the instructions of reagent manufacturers, respectively.

### Statistical analysis

All data were presented as means ± standard deviation (SD). Statistical analyses were performed with SPSS version 19.0 software (SPSS, Inc., Chicago, IL, USA), and plots were drawn by GraphPad Prism 5 software (GraphPad Software, Inc., San Diego, CA). If data are equal variances, one-way analysis of variance (ANOVA) followed by least significant difference (LSD) tests were used to compare differences among multiple groups; for the results being unequal, Dunnett’s T3 tests were taken into account. Differences with a *p* < 0.05 were considered statistically significant.

## Results

### ILG improved behavioral deficits and reduced histological damages at 24 and 72 h after ICH by intraperitoneal administration

**Fig. 1:**
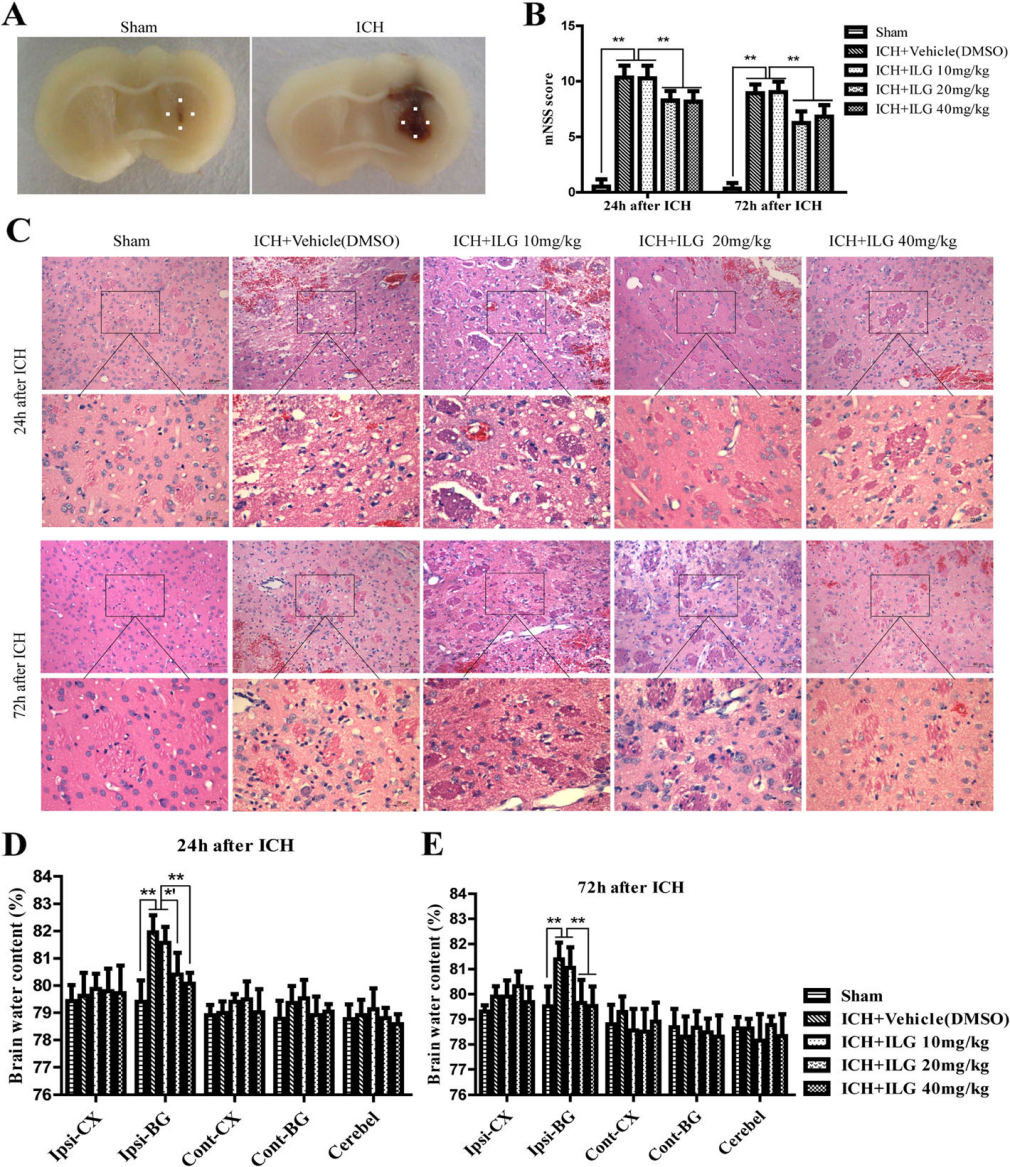
Representative macrographs and the effects of ILG treatment on ICH-induced brain impairments (**a**–**e**). Typical macrographs (*left*: sham, 24 h after operation; *right*: 24 h after ICH induction) (**a**). ILG administration at the doses of 20 and 40 mg/kg at 24 and 72 h after ICH induction significantly reduced the neurological deficits assessed by a mNSS score scale (**b**) (*n* = 12 rats/group). Similarly, ILG treatment at doses of 20 and 40 mg/kg markedly alleviated the histological changes evaluated via H&E staining (**c**) (*n* = 6 rats/group) and BWC (**d**, **e**) (*n* = 6 rats/group) measured by the dry/wet weight method at 24 and 72 h after ICH. *Scale bar* = 50 and 20 μm. Values are shown as means ± SD. **p < 0.01; *’: ICH + vehicle (DMSO) vs. ICH + ILG 20 mg/kg, *p* < 0.01; ICH + ILG 10 mg/kg vs. ICH + ILG 20 mg/kg, *p* < 0.05

Figure 1a shows the representative macrographs (left, sham, 24 h; right, ICH, 24 h). The rats subject to ICH induction showed obvious behavioral deficits at 24 and 72 h after ICH graded by a mNSS score scale (*p* < 0.01 vs. sham, 24 h and 72 h). Administration of ILG at the dosage of 10 mg/kg was not significantly effective for the improvement of behavioral deficits (vehicle-DMSO vs. 10 mg/kg: 10.33 ± 1.07 vs. 10.25 ± 1.14, *p* > 0.05, 24 h; 8.92 ± 0.79 vs. 9.00 ± 0.95, *p* > 0.05, 72 h). However, ILG obviously reduced the mNSS scores at 20 mg/kg (vehicle-DMSO vs. 20 mg/kg: vs. 8.25 ± 0.87, *p* < 0.01, 24 h; vs. 6.25 ± 1.06, *p* < 0.01, 72 h) and 40 mg/kg (vehicle-DMSO vs. 40 mg/kg: vs. 8.17 ± 0.94, *p* < 0.01, 24 h; vs. 6.83 ± 1.03, *p* < 0.01, 72 h), but the effects between two dosages were no significant difference (20 vs. 40 mg/kg, *p* > 0.05, 24 and 72 h) (Fig. 1b). Consistent with the behavioral results, H&E staining of brain tissues surrounding the hematoma showed that ILG treatment obviously improved histological impairments at the dosages of 20 and 40 mg/kg, but 10 mg/kg were not (Fig. 1c). Besides, we also evaluated the effect of ILG (20 mg/kg) on hematoma volume at 24 and 72 h after ICH induction (Additional file 1D). Typical magnetic resonance imaging (MRI) T2-weighted images (T2WI) were obtained (Additional file 2A). The hematoma volume of ICH + DMSO group at 24 and 72 h after ICH were 24.81 ± 1.64, 29.11 ± 2.06 mm^3^, respectively. ILG administration significantly reduced hematoma volume to 22.95 ± 3.26 mm^3^ at 72 h after ICH but not 24 h (23.10 ± 2.02 mm^3^) (*p* < 0.05 vs. ICH + DMSO, 72 h; *p* > 0.05 vs. ICH + DMSO, 24 h) (Additional file 2B). Consequently, the results indicated that ILG treatment (20 mg/kg) had no notably effect on bleeding but possibly promoted hematoma clearance after ICH induction.

### Intraperitoneal administration of ILG alleviated brain edema and disruption of BBB at 24 and 72 h after ICH

**Fig. 2:**
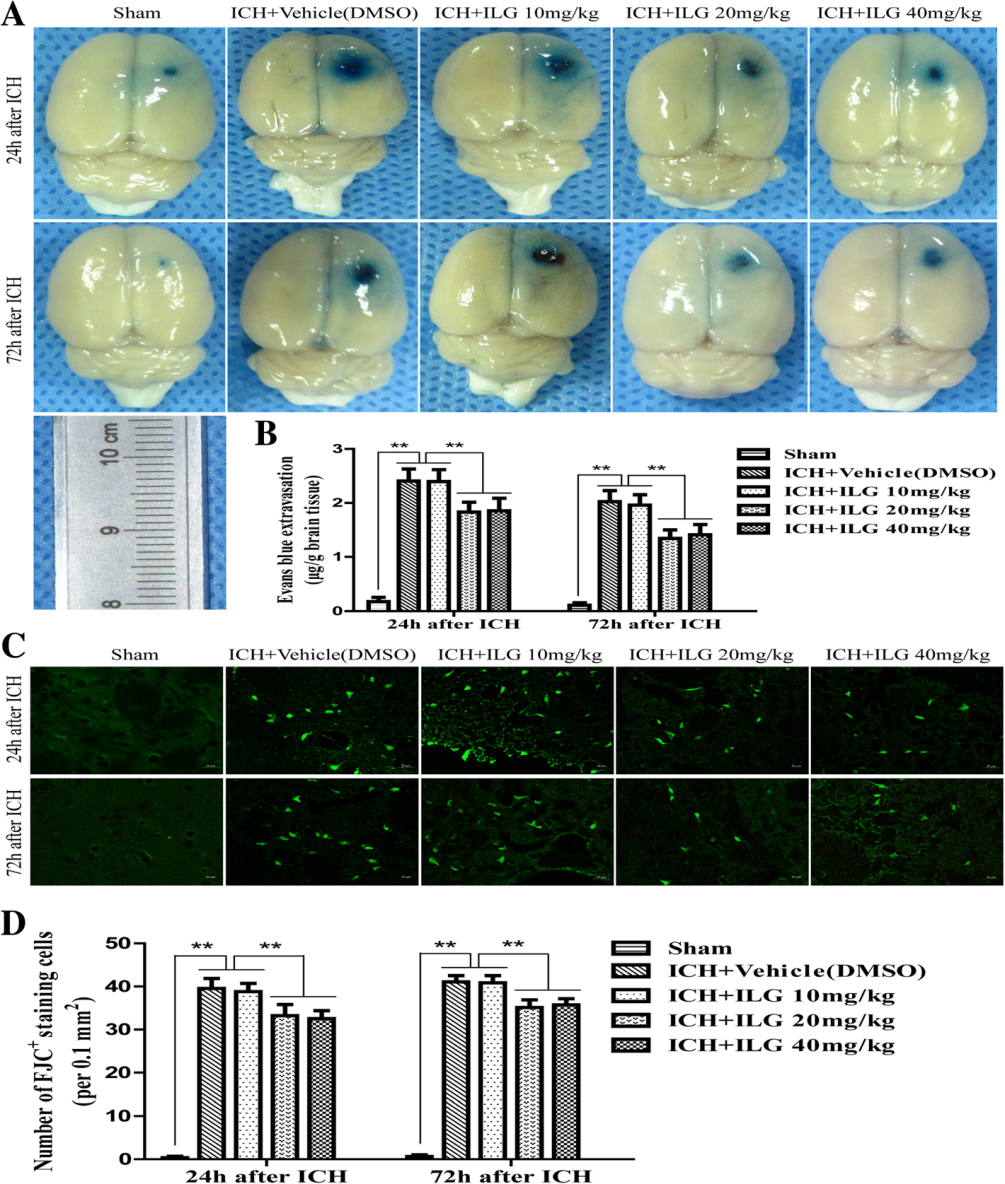
Effects of ILG on BBB disruption and the number of degenerating neuron following ICH (**a**-**d**). ILG administration at the dosages of 20 and 40 mg/kg significantly alleviated the extravasation of EB dyes and the number of FJC^+^ staining cells. Macroscopic images of brains with extravasated EB dyes (**a**) and corresponding quantitative analyses (**b**) (*n* = 6 rats/group). Typical microscopic images for FJC^+^ staining cells from injury brain tissues (**c**) and quantitative analyses of the number of FJC^+^ staining cells (**d**) (*n* = 6 rats/group). Scale bar = 20 μm. Values are reported as means ± SD. **p < 0.01

After induction of ICH at 24 and 72 h, BWC increased clearly (*p* < 0.01 vs. sham, 24 and 72 h) and Evans blue dyes significantly extravasated through disrupted BBB (*p* < 0.01 vs. sham, 24 and 72 h). The effects of ILG treatment on the brain edema and BBB disruption were in keeping with that on the behavioral deficits and brain tissue damages. Assessment of BWC using a wet/dry weight method showed that ILG treatment at the dosage of 10 mg/kg (vehicle-DMSO vs. 10 mg/kg: 81.96 ± 0.68% vs. 81.57 ± 0.64%, *p* > 0.05, 24 h; 81.40 ± 0.73% vs. 81.05 ± 0.90%, *p* > 0.05, 72 h) was unable to improve brain edema, but the dosages at 20 mg/kg (vehicle-DMSO vs. 20 mg/kg: vs. 80.40 ± 0.87%, *p* < 0.01, 24 h; vs. 79.65 ± 1.01%, *p* < 0.01, 72 h) and 40 mg/kg (vehicle-DMSO vs. 40 mg/kg: vs. 80.07 ± 0.43%, *p* < 0.01, 24 h; vs. 79.53 ± 0.85%, *p* < 0.01, 72 h) did play a positive role on the reduction of brain edema. However, there was no clear difference on the extent of protective effects about the ILG dosages of 20 and 40 mg/kg (20 vs. 40 mg/kg: *p* > 0.05, 24 and 72 h) (Fig. 1d, e). Quantitative measurements of EB dyes after extravasation in the ipsilateral hemisphere indicated that ILG treatment significantly alleviated the extravasation of EB dyes at the dosages of 20 mg/kg (*p* < 0.01 vs. vehicle-DMSO, 24 and 72 h) and 40 mg/kg (*p* < 0.01 vs. vehicle-DMSO, 24 and 72 h), but not 10 mg/kg (*p* > 0.05 vs. vehicle-DMSO, 24 and 72 h) after ICH modeling (Fig. 2a, b).

### ILG administration decreased the number of degenerating neurons in the brain tissue surrounding the hematoma after ICH induction

To further estimate the effects of ILG treatment on brain damages in a rat ICH model, we assessed the neuronal degeneration in brain tissue of perihematomal region. FJC^+^ neurons were significantly increased in the brain tissue surrounding hematoma after ICH (*p* < 0.01 vs. sham, 24 and 72 h) and administration of ILG at the dosages of 20 mg/kg (*p* < 0.01 vs. vehicle-DMSO, 24 and 72 h) and 40 mg/kg (*p* < 0.01 vs. vehicle-DMSO, 24 and 72 h) markedly dropped the number of FJC^+^ cells. Additionally, a 10 mg/kg dosage of ILG treatment had no distinct effect on the reduction of degenerating neurons (*p* > 0.05 vs. vehicle-DMSO, 24 and 72 h) and treatment results of ILG between 20 and 40 mg/kg were similar (20 vs. 40 mg/kg: *p* > 0.05, 24 and 72 h) (Fig. 2c, d).

In conclusion, experimental results displayed above clearly suggested that ILG treatment by intraperitoneal delivery significantly reduced the behavioral deficits, histological impairments, BBB disruption, brain edema and degenerating neurons of perihematomal brain tissue at the doses of 20 and 40 mg/kg, but there were no obvious difference on the extent between them. Thus, we selected the dosage of 20 mg/kg to further explore the potential molecular mechanisms of ILG’s protective effects on early brain injury after ICH induction.

### The expression and nuclear translocation of Nrf2 was promoted by ILG treatment at 24 h after ICH induction and that of NF-κB p65 was suppressed

**Fig. 3:**
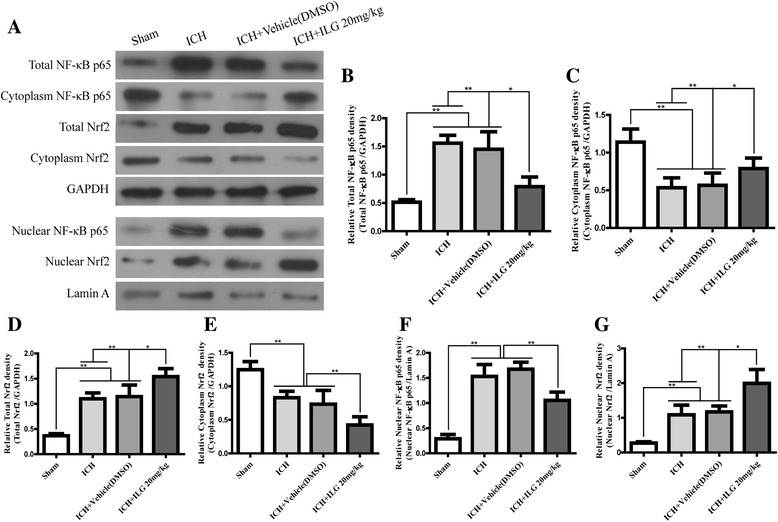
Effects of ILG on protein levels of NF-κB p65 and Nrf2 after ICH. Representative WB bands of NF-κB p65 and Nrf2 proteins (total, cytoplasmic and nuclear) (**a**) and quantitative analyses of total NF-κB p65 (**b**), cytoplasmic NF-κB p65 (**c**), and nuclear NF-κB p65 (**f**) protein levels and total Nrf2 (**d**), cytoplasmic Nrf2 (**e**), and nuclear Nrf2 (**g**) protein levels (*n* = 6 rats/group). Values are indicated by means ± SD; **p < 0.01; *p < 0.05

**Fig. 4:**
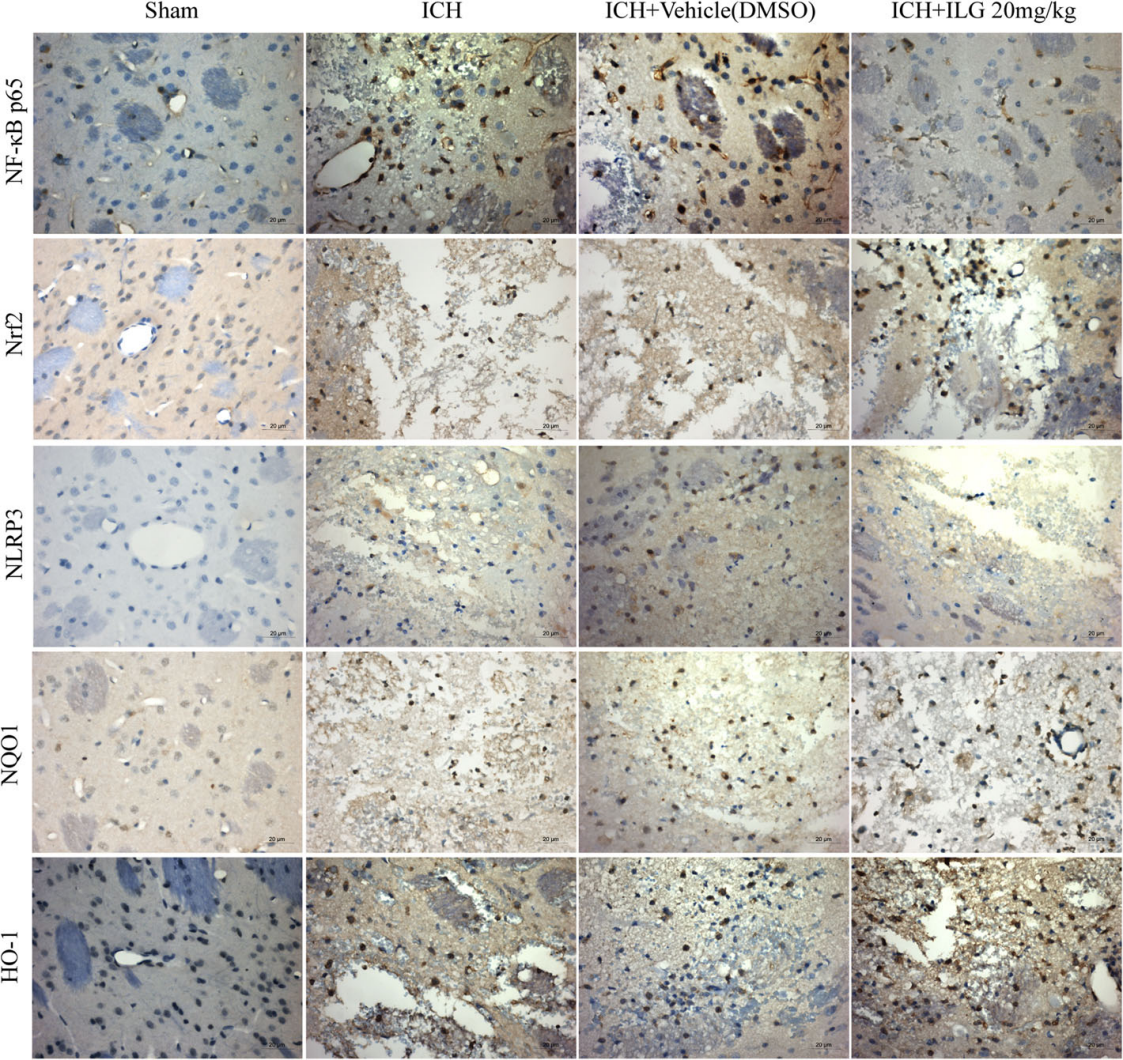
Effects of ILG on protein levels of NF-κB and Nrf2 pathways evaluated by IHC staining. Typical IHC images of NF-κB p65, Nrf2, NLRP3, NQO1, and HO-1 (*n* = 6 rats/group). *Scale bar* = 20 μm

ILG was reported to hold capacity to activate the Nrf2-mediated antioxidant system and inhibit the activation of NF-κB. Thus, we guessed that whether ILG alleviated early brain injury post ICH involved in Nrf2 and NF-κB pathways. In order to verify our supposition, we performed WB analyses and IHC staining using the perihematomal brain tissue at 24 h after ICH and the results suggested that ILG 20 mg/kg treatment significantly increased the expression of total Nrf2 protein (*p* < 0.01 vs. ICH) (Fig. 3a, d) and decreased that of total NF-κB p65 protein (*p* < 0.01 vs. ICH) (Fig. 3a, b). Typical IHC images were obtained from the injury brain tissue (Fig. 4). Meanwhile, Nrf2 protein level in the cytoplasm was significantly dropped (*p* < 0.01 vs. ICH) (Fig. 3a, e) and notably increased in the nucleus (*p* < 0.01 vs. ICH) (Fig. 3a, g); cytoplasmic level of NF-κB p65 was significantly increased (*p* < 0.01 vs. ICH) (Fig. 3a, c) and the nuclear level was notably deceased (*p* < 0.01 vs. ICH) (Fig. 3a, f). Consequently, experimental results indicated that ILG promoted the expression and nuclear translocation of Nrf2 and suppressed that of NF-κB p65.

### The components of NLRP3 inflammasome pathway and subsequent IL-1β/IL-18 release were suppressed by ILG treatment

**Fig. 5:**
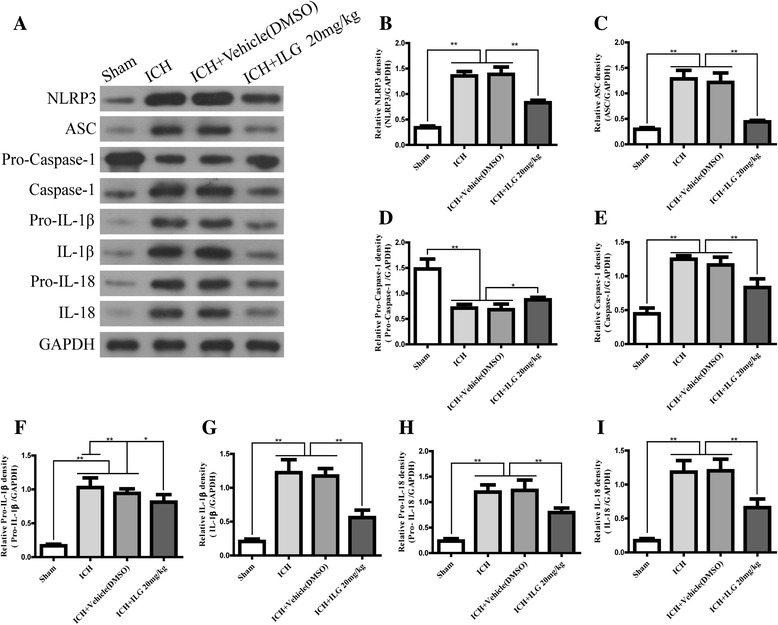
Effects of ILG on NLRP3 inflammasome activation and IL-1β/IL-18 maturation. Representative WB bands (**a**) and inhibited effects of ILG on NLRP3 (**b**), ASC (**c**), pro-caspase-1 (**d**), caspase-1 (**e**), pro-IL-1β (**f**), IL-1β (**g**), pro-IL-18 (**h**), and IL-18 (**i**) protein levels in the ipsilateral hemisphere at 24 h after ICH (*n* = 6 rats/group). Data are indicated by means ± SD. *p < 0.05; **p < 0.01

Activation of NLRP3 inflammasome and induction of its components aggravated the early brain injury after ICH, and blockades were protective reported by our [17] and other studies [8, 16]. In addition, a recent study showed that Nrf2 negatively regulates NLRP3 inflammasome activity by inhibiting ROS [18]. Therefore, we investigated that whether ILG decreased the activation and induction of NLRP3 inflammasome pathway components or not. The results showed that ILG at the dosage of 20 mg/kg significantly suppressed the expression of NLRP3 inflammasome components: NLRP3, ASC, caspase-1 (*p* < 0.01 vs. ICH) (Fig. 5a, b, c, e), and blocked the activation of NLRP3 inflammasome as indicated by the reduction of cleaved IL-1β and IL-18 (*p* < 0.01 vs. ICH) (Fig. 5a, g, i). Representative IHC images of NLRP3 protein were shown (Fig. 4). Consistent with the above results, ILG treatment obviously reduced the protein levels of pro-IL-1β (*p* < 0.01 vs. ICH) (Fig. 5a, f) and pro-IL-18 (*p* < 0.01 vs. ICH) (Fig. 5a, h) and increased the expression of pro-caspase-1 (*p* < 0.05 vs. ICH) (Fig. 5a, d).

### ILG delivery reduced the markers of oxidative stress injury, infiltration of MPO^+^, CD68^+^, Iba-1^+^ cells, and expression of MPO in the brain tissue surrounding the hematoma

**Fig. 6:**
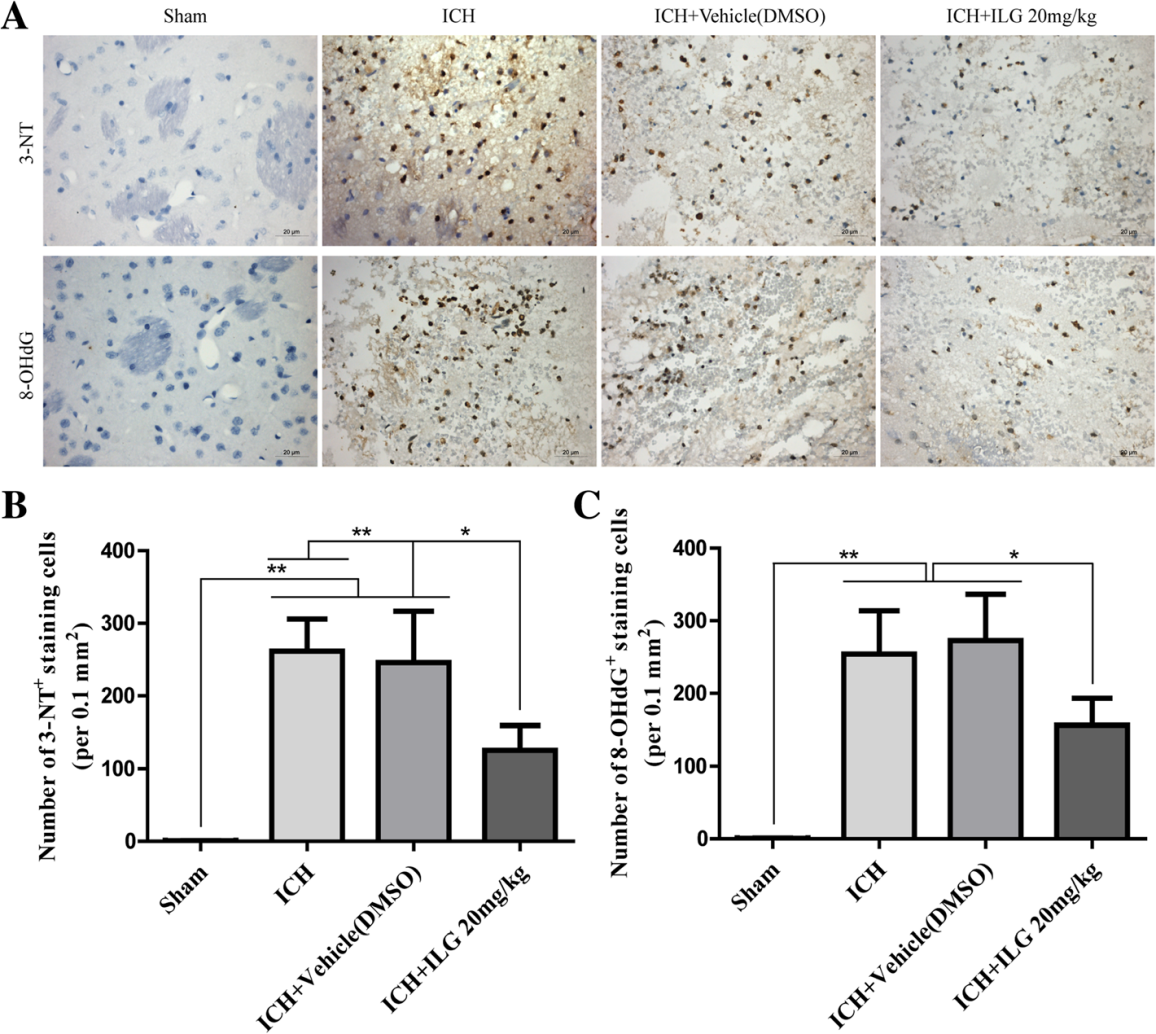
Effects of ILG on oxidative stress marker levels in the injury brain tissue after ICH. ILG significantly decreased the oxidative stress marker levels (3-NT and 8-OHdG) after ICH. Typical oxidative stress markers 3-NT and 8-OHdG IHC images (**a**) and quantitative analyses (**b**, **c**) (*n* = 6 rats/group). *Scale bar* = 20 μm. Values are reported as means ± SD. **p < 0.01; *p < 0.05

**Fig. 7:**
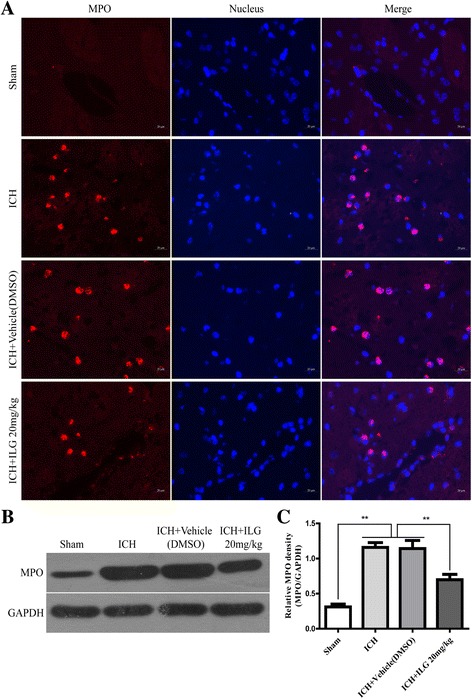
Effects of ILG on the number of MPO^+^ cells in perihematomal brain tissues (**a**–**c**). Representative microscopic images (**a**), WB bands (**b**), and quantitative analysis of the bands (**c**) (*n* = 6 rats/group). *Scale bar* = 20 μm. Values are reported as means ± SD. **p < 0.01

Excessive production of ROS mediates seriously oxidative injury and is a key promoting factor for the activation of NLRP3 inflammasome, and the antioxidant responses initiated through nuclear translocation of Nrf2 could alleviate the ROS-induced brain injury and inflammatory cell infiltration. Therefore, we probed that if ILG treatment at 20 mg/kg dropped the production of oxidative stress markers 3-NT and 8-OHdG by IHC staining and infiltration of neutrophils (indicated by MPO, a neutrophil marker) using IF staining and WB analyses. The results suggested that ILG (20 mg/kg) could significantly decrease the amounts of 3-NT^+^ cells (*p* < 0.01 vs. ICH) (Fig. 6a, b), 8-OHdG^+^ cells (*p* < 0.05 vs. ICH) (Fig. 6a, c) in perihematomal brain tissues. Also, the expression level of MPO was notably decreased in the injury brain tissue (*p* < 0.01 vs. ICH) (Fig. 7c), and representative IF images and WB band of MPO protein were shown, respectively (Fig. 7a, b). Meanwhile, ILG (20 mg/kg) could significantly drop the amounts of CD68^+^, Iba-1^+^ (CD68 and Iba-1, the microglia/macrophage markers) cell recruitments in injury brain tissue after ICH as well (*p* < 0.05 vs. ICH) (Additional file 2C, D, E).

### ILG treatment lowered the mRNA levels of NLRP3 inflammasome, NF-κB, and Nrf2 pathway components

**Fig. 8:**
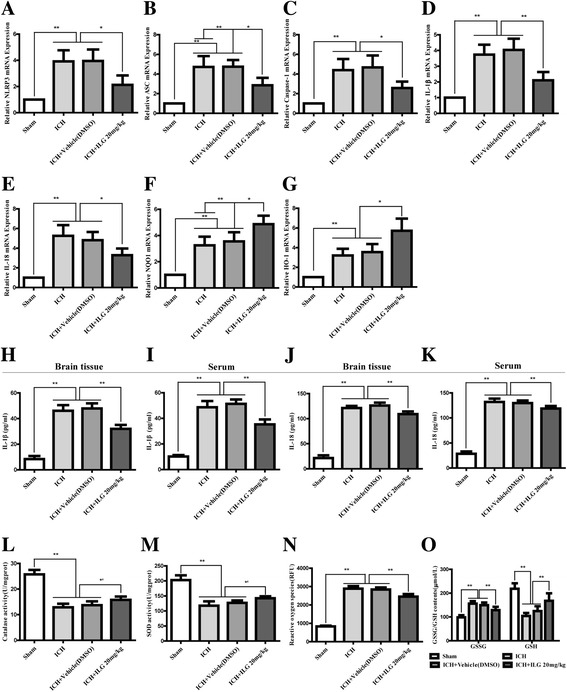
Effects of ILG on the mRNA levels of NLRP3 inflammasome pathway components and downstream molecules of Nrf2-mediated antioxidant system, contents of inflammatory cytokines and antioxidants, and activity of antioxidative enzymes. ILG treatment at 20 mg/kg notably decreased the mRNA levels of NLRP3 (**a**), ASC (**b**), caspase-1 (**c**), IL-1β (**d**), IL-18 (**e**), and further increased the NQO1 (**f**), HO-1 (**g**) mRNA levels (*n* = 6 rats/group). Similarly, ILG administration at 20 mg/kg obviously reduced the levels of IL-1β (**h**, **i**) and IL-18 (**j**, **k**) in the perihematomal brain tissue and serum measured by ELISA (*n* = 6 rats/group). Besides, ILG delivery also markedly reversed the reduction of CAT and SOD activities (**l**, **m**), increasing of ROS (**n**) and GSSG (**o**) contents and decreasing of GSH level (**o**) (*n* = 6 rats/group). Values are reported as means ± SD. **p < 0.01; *p < 0.05; *’: ICH vs. ICH + ILG 20 mg/kg, *p* < 0.01; ICH + vehicle (DMSO) vs. ICH + ILG 20 mg/kg, *p* < 0.05

To further explore the effects of ILG treatment on the mRNA levels of NLRP3 inflammasome, NF-κB, and Nrf2 pathway components at 24 h after ICH induction, we performed relative real-time RT-qPCR study. The results showed that 24 h after ICH induction, the mRNA levels of NLRP3, ASC, caspase-1, IL-1β, IL-18, NQO1, and HO-1 were obviously increased (*p* < 0.01 vs. sham) (Fig. 8) and ILG (20 mg/kg) significantly weakened the increases of NLRP3, caspase-1, IL-18 (*p* < 0.05 vs. ICH) (Fig. 8a, c, e), ASC, and IL-1β mRNA levels (*p* < 0.01 vs. ICH) (Fig. 8b, d). Meanwhile, NQO1 (*p* < 0.01 vs. ICH) (Fig. 8f) and HO-1 (*p* < 0.05 vs. ICH) (Fig. 8g) were further distinctly upregulated at the mRNA levels by ILG treatment. In addition, representative IHC images of NQO1 and HO-1 proteins were shown (Fig. 4).

### Inflammatory cytokine levels in the brain tissue and serum from the blood of cardiac puncture were both significantly reduced by ILG treatment

We measured the levels of inflammatory cytokines IL-1β and IL-18 both in perihematomal brain tissues and the serum, respectively. The results were similar to the previous experimental findings, namely, ILG treatment significantly dropped the levels of IL-1β in damaged brain tissues and the serum (*p* < 0.01 vs. ICH) (Fig. 8h, i). The contents of IL-18 in the damaged brain tissue and serum were significantly decreased as well (*p* < 0.01 vs. ICH) (Fig. 8j, k).

### ILG reduced the contents of ROS and GSSG, increased the level of GSH and upregulated the enzyme activities of SOD and CAT

We also detected the activities of SOD and CAT enzymes and the contents of GSH/GSSG and ROS after ILG treatment at 24 h post ICH. Experimental results indicated that after ICH induction, enzyme activities of CAT and SOD and the content of GSH were significantly decreased (*p* < 0.01 vs. sham) (Fig. 8l, m, o), the levels of GSSG and ROS were evidently increased (*p* < 0.01 vs. sham) (Fig. 8o, n). ILG treatment at 20 mg/kg could strikingly reverse the decreases of CAT and SOD enzyme activities, increases of GSSG and ROS levels, and reduction of GSH content (*p* < 0.01 vs. ICH) (Fig. 8l–o).

### Nrf2 siRNA interference aggravated the behavioral deficits and brain edema and raised the number of FJC^+^ cells and administration of ILG lowered those uncomfortable effects

**Fig. 9:**
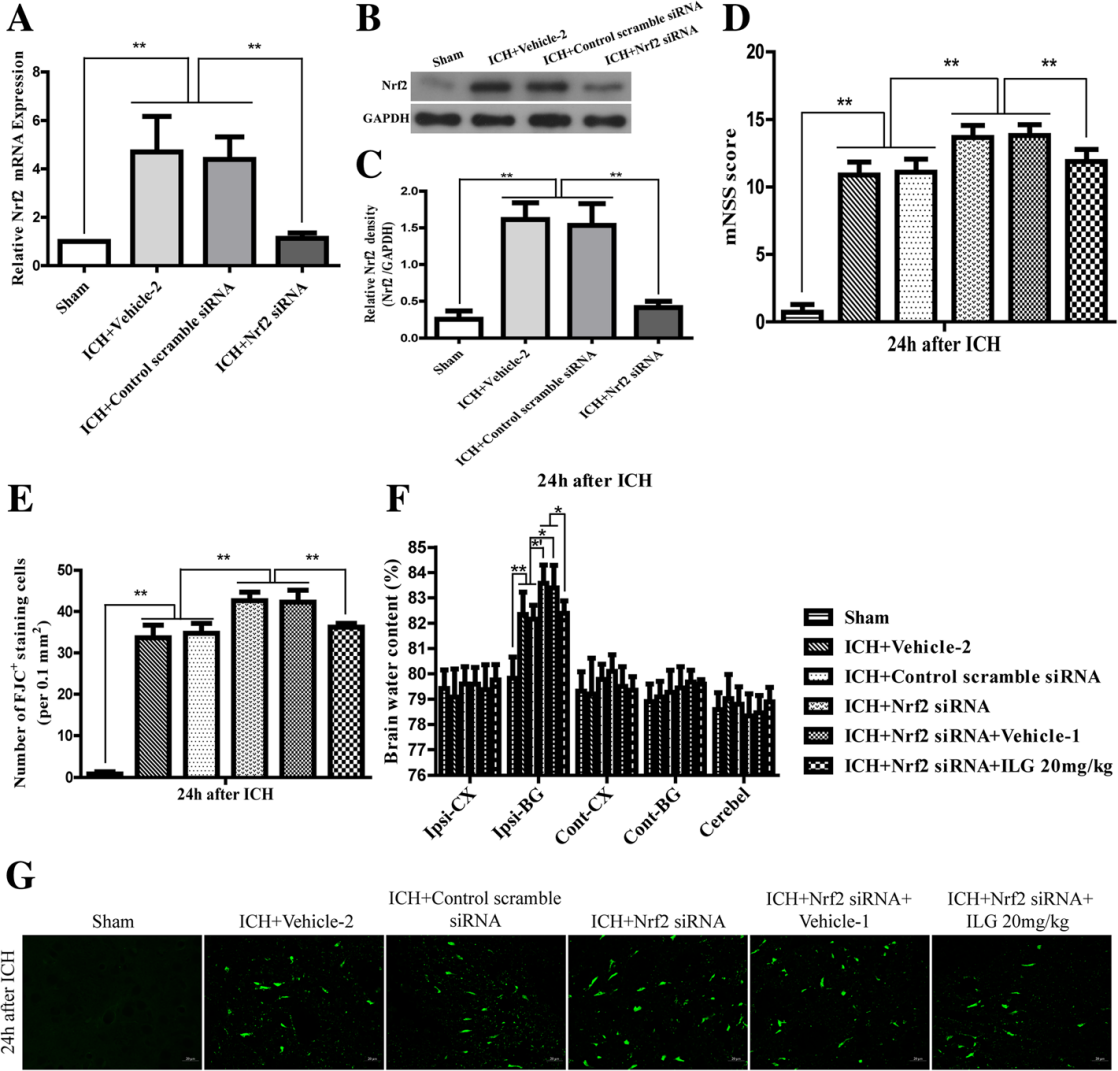
Effects of Nrf2 siRNA delivery and Nrf2 siRNA with ILG 20 mg/kg co-administration in ICH rats. Real-time RT-qPCR assay of Nrf2 after siRNA delivery 24 h following ICH (*n* = 6 rats/group) (**a**). WB assay (**b**) and quantification (**c**) of Nrf2 protein after siRNA treatment 24 h following ICH (*n* = 6 rats/group); mNSS score (**d**) at 24 h post ICH (*n* = 18 rats/group). BWC (**f**) at 24 h after ICH (*n* = 6 rats/group). FJC staining (**g**) and quantification (**e**) of FJC^+^ staining cells (*n* = 6 rats/group). Data represent means ± SD. *Scale bar* = 20 μm. **p < 0.01; *p < 0.05. *’: ICH + vehicle-2 vs. ICH + Nrf2 siRNA, *p* < 0.05; ICH + control scramble siRNA vs. ICH + Nrf2 siRNA, *p* < 0.01

In our studies mentioned above, some preliminary conclusions were obtained that ILG could effectively reduce the early brain injury after ICH induction and the protective effects of ILG might be involved in the regulation of Nrf2, ROS, NF-κB, and NLPR3 inflammasome pathways. We wondered if ILG treatment reduced the brain injury mediated by NLRP3 inflammasome pathway via Nrf2 activation-induced ROS and/or NF-κB inhibition. We conducted the Nrf2 siRNA interfering and Nrf2 siRNA together with ILG 20 mg/kg co-treatment research by intraventricular injection and intraperitoneal delivery, respectively. Interfering effects of Nrf2 siRNA were identified using real-time RT-qPCR and WB analyses. The results showed that Nrf2 siRNA significantly dropped the mRNA and protein expression (*p* < 0.01 vs. vehicle-2) levels of Nrf2 (Fig. 9a–c). In the study, we found that Nrf2 siRNA interference markedly exacerbated the function deficits (Nrf2 siRNA vs. vehicle-2: 13.67 ± 0.91 vs. 10.89 ± 0.96, *p* < 0.01) (Fig. 9d) and brain edema (Nrf2 siRNA vs. vehicle-2: 83.57 ± 0.80% vs. 82.35 ± 0.98%, *p* < 0.05) (Fig. 9f) and increased the amounts of degenerating neuronal cells (*p* < 0.01 vs. vehicle-2) (Fig. 9e, g) at 24 h after ICH induction. However, ILG administration (20 mg/kg) distinctly reversed those uncomfortable results: function deficits (Nrf2 siRNA vs. Nrf2 siRNA + ILG 20 mg/kg: vs. 11.89 ± 0.90, *p* < 0.01), brain edema (Nrf2 siRNA vs. Nrf2 siRNA + ILG 20 mg/kg: vs. 82.27 ± 0.57%, *p* < 0.05), and FJC^+^ cells (*p* < 0.01 vs. Nrf2 siRNA) (Fig. 9d–g).

### Nrf2 siRNA interference increased the expression of NF-κB p65 and NLRP3 inflammasome components and triggered the activation of NLRP3 inflammasome pathway and ILG reduced these effects

**Fig. 10:**
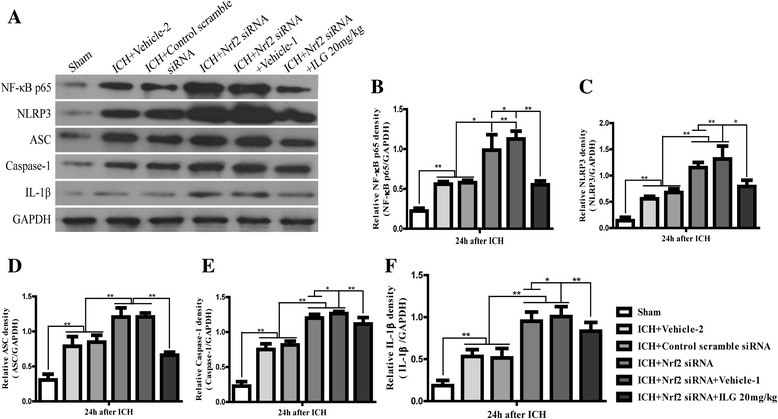
Effects of Nrf2 siRNA and Nrf2 siRNA with ILG co-treatment on the protein levels of NF-κB p65 and NLRP3 inflammasome pathway components after ICH. WB assay (**a**) and quantification of NF-κB p65 (**b**), NLRP3 (**c**), ASC (**d**), caspase-1 (**e**), and IL-1β (**f**) protein levels after Nrf2 siRNA and ILG 20 mg/kg co-treatment at 24 h following ICH (*n* = 6 rats/group). Data are indicated by means ± SD. **p < 0.01; *p < 0.05

We further evaluated the effects of Nrf2 siRNA on the expression of NF-κB p65 and induction and activation of NLRP3 inflammasome pathway components. We found that Nrf2 siRNA interfering could significantly increase the expression of NF-κB p65 (*p* < 0.05 vs. vehicle-2) (Fig. 10a, b); NLRP3 inflammasome components: NLRP3, ASC, caspase-1, and downstream molecule, IL-1β (*p* < 0.01 vs. vehicle-2) (Fig. 10a, c, d, e, f). ILG at the dosage of 20 mg/kg and Nrf2 siRNA co-administration obviously alleviated the enhancement of protein expression levels of NF-κB p65, caspase-1, IL-1β (*p* < 0.05 vs. Nrf2 siRNA) (Fig. 10a, b, e, f), NLRP3, and ASC (*p* < 0.01 vs. Nrf2 siRNA) (Fig. 10a, c, d) after Nrf2 siRNA treatment at 24 h following ICH.

## Discussion

Accumulating studies have displayed that oxidative stress and inflammation played key roles in the pathophysiological processes of early brain injury after ICH induction and inhibition of them were beneficial [5, 7, 44–46]. In our first experiment, we found that ILG administration at the dosages of 20 and 40 mg/kg ameliorated the early brain tissue impairments and behavioral defects as indicated by the reduction of mNSS scores and FJC^+^ neuronal cells, the improvement of histological damages, BBB disruption, and brain edema at 24 and 72 h post ICH modeling and obtained the ideal dose of ILG at 20 mg/kg for the following experiments. In the second experiment, the results showed that ILG delivery at 20 mg/kg activated the Nrf2-mediated antioxidative signaling pathway and suppressed the activation of NF-κB and NLRP3 inflammasome pathways as indicated by the increasing of nuclear translocation and decreasing of the cytoplasmic level of Nrf2, the reduction of nuclear translocation and upregulation of cytoplasmic level of NF-κB p65, and the induction and activation of NLRP3 inflammasome (components) and its downstream molecules. In the third experiment, we found that Nrf2 siRNA notably exacerbated the early brain injury post ICH as supported by aggravated behavioral deficits, brain edema, and degeneration of neuronal cells, and ILG treatment visibly alleviated the effects. Based on the results above, we hypothesized here that ILG alleviates early brain injury after ICH induction by activating Nrf2 antioxidant pathway, inhibiting the activation and induction of NLRP3 inflammasome (components), and this process may be involved in the suppression of ROS and/or NF-κB signaling pathways. Potential molecular mechanisms of ILG’s effects on the early brain injury after ICH induction is shown in Additional file 3.

Results from our study have powerful evidence showing that ILG possesses a brain cell-protective function, and this was in line with previous studies in vivo [47–50] and in vitro [26, 51, 52]. Pretreatment of ILG significantly alleviated neurological deficits, cerebral infarct, and brain edema, and these neuroprotective effects are involved in the increases of brain ATP content, energy charge (EC) and total adenine nucleotides (TAN) and preservation of brain Na^+^ K^+^ ATPase activity, SOD, CAT, and GSH-Px, and inhibition of the increase of brain MDA content in a rat cerebral ischemia-reperfusion model [50]. Toxicity of brain cells induced by cocaine and methamphetamine delivery was also able to be attenuated by ILG treatment [47–49]. Consistently, in in vitro studies, ILG protected neuronal cells from glutamate and 6-hydroxydopamine (6-OHDA)-induced neurotoxicity by reducing the production of ROS [51, 52]. Meanwhile, a recent report also showed that ILG treatment could notably alleviate rotenone and sodium nitroprusside (SNP)-induced oxidative stress and nitrosative stress by improving MMP, ATP levels, and neural cell viability [26].

ILG, one of the active extracts isolated from *G. uralensis*, is a flavonoid with chalcone structure and is brain-permeable after administration [53], which showed various biological activities including anti-inflammatory, anti-oxidative stress [9]. Increasing studies suggested that ILG exerts biological effects by activating Nrf2-mediated anti-oxidative system and eliminating ROS [9] and was the most potent inducer to stimulate the expression of Nrf2 and its downstream genes [28]. The activation mechanisms of Nrf2 by ILG might be involved in the alkylation of kelchlike ECH-associated protein 1 (Keap1) at specific cysteine residues, especially at the site of C151, a most reactive cysteine residue site of human Keap1 [54]. At the same time, there were reports showing that ILG upregulated the expression of HO-1 in RAW264.7 macrophages through the extracellular signal-regulated kinase1/2 (ERK1/2) pathway post lipopolysaccharide (LPS) treatment [55] and had inhibitory effects on LPS-induced inflammatory responses of mouse macrophages by suppressing NF-κB signaling involved in the blockade of inhibitor of κBα (IκBα) degradation and phosphorylation [56]. In addition, recent reports also indicated that ILG induced the activation of Nrf2 as indicated by an increase in its nuclear translocation and the expression of Nrf2-targeted phase II enzymes, such as HO-1 and NQO1 [29]. In addition to the regulation of ILG on Nrf2 pathway, increasing evidences have suggested that ILG notably inhibited the activation of NF-κB pathway by suppressing LPS-induced TLR4/MD-2 homodimerization [57], blocking IκBα phosphorylation and degradation, reducing NF-κB p65 nuclear translocation [58], downregulating mRNA and protein levels of NF-κB and its activation [25, 59], and inhibiting RANKL-stimulated NF-κB expression and nuclear translocation [23].

The NLRP3 inflammasome, a best characterized pattern recognition receptor (PRR) in innate immune response, played a crucial component in the early brain injury post ICH [8, 16, 17] and was composed by a sensor (NLRP3 protein), an adaptor (ASC protein), and an effector (zymogen pro-caspase-1) [60, 61]. Production of ROS, mitochondrial DNA or the mitochondrial phospholipid cardiolipin, potassium efflux, changes in cell volume, calcium, and lysosomal impairments have all been proposed as critical active signals to trigger the activation of NLRP3 inflammasome [60, 61]. Activation of NLRP3 inflammasome demands two signals. One is the stimulus of NF-kB pathway that after NF-κB activation, NF-κB translocates into the nucleus, after binding with DNA and initiating the transcription and translation of IL-1β precursor protein and NLRP3 protein; another one is to trigger the assembly of NLRP3 inflammasome and lead to its stimulus such as production of ROS. Following triggered, active NLRP3 inflammasome recruits precursor caspase-1 and cleaves it into active caspase-1, and then the cleaved caspase-1 processes IL-1β and IL-18 precursors into mature IL-1β and IL-18, eventually augmenting the inflammatory responses and impairments [60, 61].

Recently, several studies have demonstrated that NLRP3 inflammasome pathway was activated after ICH induction, and the inhibiting of NLRP3 using siRNA or recombinant adenovirus encoding NLRP3 RNAi could attenuate the brain injury including improving behavioral deficits and reducing brain edema and MPO level. Hence, ROS and the activation of NF-κB pathway were the crucial upstream signals, and blocking them can reduce the activation of NLRP3 inflammasome. In our study, we have verified that Nrf2 triggering via ILG administration significantly decreased the production of oxidative stress markers 3-NT and 8-OHdG and suppressed the activation of NF-κB; meanwhile, the NLRP3 inflammasome was restrained. The results suggested that Nrf2 could downregulate the activity and expression of NLRP3 inflammasome (components) and were in keeping with previous reports [18, 22]. To further proved our assumption, we conducted Nrf2 siRNA interfering and Nrf2 siRNA + ILG 20 mg/kg co-administration study in a rat ICH model. The results showed that after Nrf2 siRNA treatment, the brain injuries were more severe and ILG (20 mg/kg) obviously attenuated the impairments. These also were consistent with the studies of other groups, namely, Nrf2 was involved in the regulation of ILG-mediated NLRP3 inflammasome activation [18, 22] and the activation of Nrf2 pathway was neuroprotective [9, 12, 13, 31].

Microglia are the key immune cells existing in the central nervous system (CNS) and are commonly referred to as the macrophage of the brain. Microglia are considered to be the first non-neuronal cells to react to various pathological processes after ICH and thus once the condition ictus, microglia are immediately activated by a various of blood components and then activated microglia release multiple cytokines and chemokines, following peripheral inflammatory cells (including neutrophils, macrophages) infiltrating into the hemorrhagic brain and are activated, next producing a mass of cytokines, chemokines, and cytotoxicity substance [4, 62]. Thus, both the activation and infiltration of neutrophils and microglia/macrophages synergistically contribute to the inflammatory brain injury post ICH and play crucial role on the pathological mechanisms [4, 62]. Our experimental results have shown that ILG administration could notably reduce the number of perihematomal neutrophils and microglia/macrophages as well.

Also, iron, one key component of hemoglobin (Hb) metabolites, was reported to be involved in the secondary brain injury. Excessive production of iron could lead to oxidative brain impairments by Fenton reaction, which yields massive ROS. Furthermore, HO-1 promotes the level of iron by participating in the degradation of heme [63, 64]. Besides, ILG was also reported to counteract iron-catalyzed oxidative stress damages in HepG2 cells by AMPK-mediated GSK3β inhibition which was involved in mitochondrial dysfunction and superoxide generation [65]. Meanwhile, in our experiments, we found that ILG administration could significantly inhibit the level of ROS and promote the expression of HO-1 in the injury brain tissue. Thus, the relationship of iron metabolism and neuroprotective effects of ILG needs to be further explored.

Thrombin, a serine protease produced immediately in brain after ICH to prevent the bleeding. It may be a central injury mechanism of ICH and was shown to mediate the secondary brain injury by multiple pathways including complement cascade and protease-activated receptors (PAR) [63, 64]. Recently, thrombin was also reported to be involved in the activation of NLRP3 inflammasome and microglia and induced severe brain injury including brain edema, BBB disruption, and brain cell loss [66, 67]. In our study, we found that ILG 20 mg/kg could efficaciously blockade the activation of NLRP3 inflammasome and infiltration of neutrophils and microglia/macrophages into the injury brain tissue. Thus, whether ILG exerts its protective effects involved in the thrombin-mediated brain injury pathway remains to be further investigated.

There are several potential limitations deserving attention in our experiments. Firstly, ILG was reported to perform a variety of biological functions by regulating multiple signaling pathways, and we mainly concentrated on the Nrf2, ROS, NF-κB, and NLRP3 inflammasome pathways. However, ILG may exert its effects on other signaling pathways. Secondly, we just carried out our study on a kind of ICH model (sterile-filtered collagenase type IV-induced), not concurrently on other ICH models such as autologous blood-imitated model of ICH. The results derived from our experiments should be more convincing by more than one model to be verified. Finally, collagenase itself is a kind of foreign matter and is unavoidable to trigger extra inflammatory responses. Consequently, further experiments are needed to settle these issues.

## Conclusions

Taken together, our experiments have identified that ILG administration notably attenuated the early brain injury after ICH induction and the underlying molecular mechanisms of these beneficial effects are involved in the regulation of ROS and/or NF-κB on the activation of NLRP3 inflammasome pathway by the triggering of Nrf2 activity and Nrf2-induced antioxidant system. In addition, our experimental results might provide a novel therapeutical strategy for ICH.

## Additional files

Additional file 1: Experimental design and groups (a-c). MRI and Hematoma Volume Evaluation (d). The captions of Additional file 2 and 3 (e). (PDF 235 kb)

Additional file 2: Effects of ILG on the hematoma volume and expansion at 24 h and 72 h after ICH (a, b) and effects of ILG on the number of CD68^+^, Iba-1^+^ cells in the perihematomal brain tissue at 24 h after ICH (c-e). Representative MRI T2WI images (a) and quantitative analyses of hematoma volume (b) (*n* = 6 rats / group). Representative microscopic images (c) and quantitative analyses of CD68^+^, Iba-1^+^ cells (d, e) (*n* = 6 rats /group). Scale bar = 20 μm. Values are reported as means ± SD. ** *p* < 0.01, * *p* < 0.05. (TIF 7318 kb)

Additional file 3: Mechanism diagram. Underlying molecular mechanisms of ILG’s neuroprotective effects on the early brain injury after ICH induction. ILG alleviated the early brain injury following ICH may be involved in the regulation of ROS and / or NF-κB on the activation of NLRP3 inflammasome pathway by the triggering of Nrf2 activity and the induction of Nrf2-mediated antioxidant system. (TIF 232 kb)

## Abbreviations

3-NT: 3-Nitrotyrosine
6-OHDA: 6-Hydroxydopamine
8-OHdG: 8-Hydroxyguanosine
ANOVA: Analysis of variance
ARE: Antioxidant response element
BBB: Blood-brain barrier
BSA: Bovine serum albumin
BWC: Brain water content
CAT: Catalase
CNS: Central nervous system
DAB: 3,3-Diaminobenzidine
DAPI: 4′6-Diamidino-2-phenylindole
DMSO: Dimethylsulfoxide
EB: Evans blue
EC: Energy charge
EDTA: Ethylene diamine tetraacetic acid
ELISA: Enzyme-linked immunosorbent assay
ERK1/2: Extracellular signal-regulated kinase1/2
FJC: Fluoro-Jade® C
*G. uralensis*: *Glycyrrhiza uralensis*
GAPDH: Glyceraldehyde 3-phosphate dehydrogenase
GPX: Glutathione peroxidase
GSH/GSSG: Glutathione/oxidized glutathione
GST: Glutathione-S-transferase
H&E: Hematoxylin and eosin
Hb: Hemoglobin
HO-1: Heme oxygenase-1
HRP: Horseradish peroxidase
i.p.: Intraperitoneal injection
ICH: Intracerebral hemorrhage
IF: Immunofluorescence
IHC: Immunohistochemistry
IL-1β: Interleukin-1 beta
ILG: Isoliquiritigenin
IκBα: Inhibitor of κBα
Keap1: Kelchlike ECH-associated protein 1
LPS: Lipopolysaccharide
LSD: Least significant difference
mNSS: Modified Neurological Severity Score
MPO: Myeloperoxidase
MRI: Magnetic resonance imaging
NF-κB: Nuclear factor-κB
NLRP3: Nod-like receptor family, pyrin domain-containing 3
NO: Nitric oxide
NQO1: NAD(P)H: quinone oxidoreductase-1
Nrf2: Nuclear factor erythroid-2 related factor 2
PAR: Protease-activated receptor
PBS: Phosphate buffer solution
PRR: Pattern recognition receptor
ROS: Reactive oxygen species
RT-qPCR: Real-time reverse transcription-quantitative polymerase chain reaction
SD: Standard deviation
SD rats: Sprague-Dawley rats
siRNA: Small interfering RNA
SNP: Sodium nitroprusside
SOD: Superoxide dismutase
SPF: Specific pathogen-free
T2WI: T2-weighted images
TAN: Total adenine nucleotides
WB: Western blot
