# Supplementary material for: Isoliquiritigenin alleviates early brain injury after experimental intracerebral hemorrhage via suppressing ROS- and/or NF-κB-mediated NLRP3 inflammasome activation by promoting Nrf2 antioxidant pathway
Source: J Neuroinflammation. 2017 Jun 13;14:119. doi: 10.1186/s12974-017-0895-5 (PMC5470182; doi:10.1186/s12974-017-0895-5)
Supplement: Supplementary file 1 — Experimental design and groups (a-c). MRI and Hematoma Volume Evaluation (d). The captions of Additional file 2 and 3 (e). (PDF 235 kb) [file 12974_2017_895_MOESM1_ESM.pdf]

A:

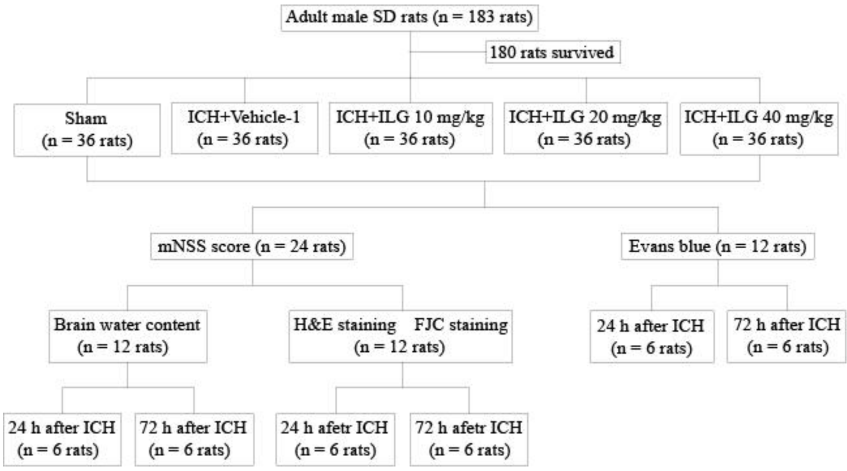
Adult male SD rats (n = 183 rats)
180 rats survived
Sham (n = 36 rats)
ICH+Vehicle-1 (n = 36 rats)
ICH+ILG 10 mg/kg (n = 36 rats)
ICH+ILG 20 mg/kg (n = 36 rats)
ICH+ILG 40 mg/kg (n = 36 rats)
mNSS score (n = 24 rats)
Evans blue (n = 12 rats)
Brain water content (n = 12 rats)
H&E staining FJC staining (n = 12 rats)
24 h after ICH (n = 6 rats)
72 h after ICH (n = 6 rats)
24 h after ICH (n = 6 rats)
72 h after ICH (n = 6 rats)
24 h afetr ICH (n = 6 rats)
72 h afetr ICH (n = 6 rats)

B:

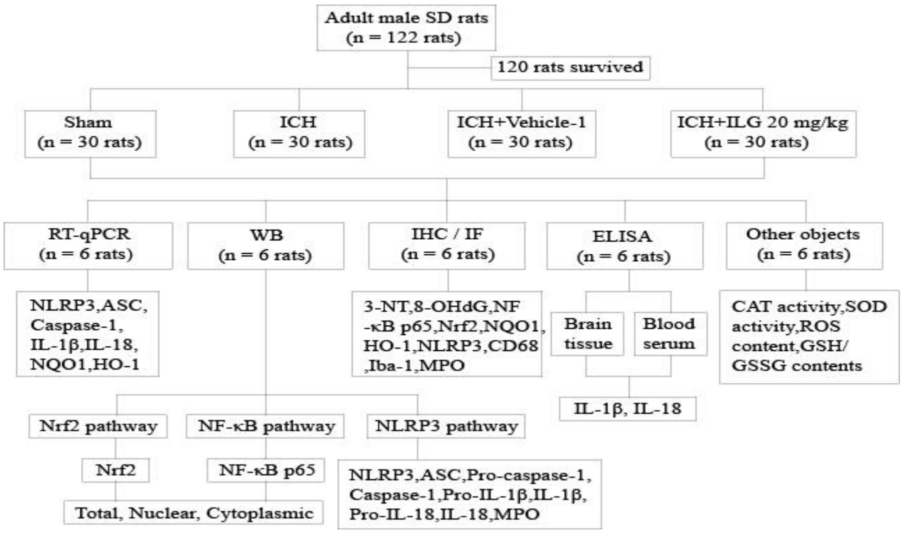
Adult male SD rats (n = 122 rats)
120 rats survived
Sham (n = 30 rats)
ICH (n = 30 rats)
ICH+Vehicle-1 (n = 30 rats)
ICH+ILG 20 mg/kg (n = 30 rats)
RT-qPCR (n = 6 rats)
WB (n = 6 rats)
IHC / IF (n = 6 rats)
ELISA (n = 6 rats)
Other objects (n = 6 rats)
NLRP3,ASC, Caspase-1, IL-1β,IL-18, NQO1,HO-1
3-NT,8-OHdG,NF -κB p65,Nrf2,NQO1, HO-1,NLRP3,CD68 ,Iba-1,MPO
Brain tissue
Blood serum
IL-1β, IL-18
CAT activity,SOD activity,ROS content,GSH/ GSSG contents
Nrf2 pathway
NF-κB pathway
NLRP3 pathway
Nrf2
NF-κB p65
Total, Nuclear, Cytoplasmic
NLRP3,ASC,Pro-caspase-1, Caspase-1,Pro-IL-1β,IL-1β, Pro-IL-18,IL-18,MPO

C:

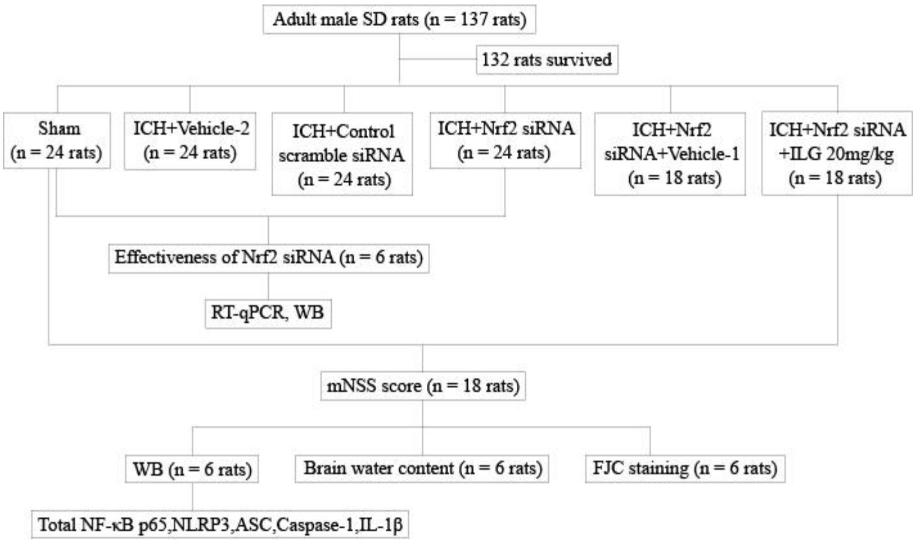
Adult male SD rats (n = 137 rats)
132 rats survived
Sham (n = 24 rats)
ICH+Vehicle-2 (n = 24 rats)
ICH+Control scramble siRNA (n = 24 rats)
ICH+Nrf2 siRNA (n = 24 rats)
ICH+Nrf2 siRNA+Vehicle-1 (n = 18 rats)
ICH+Nrf2 siRNA +ILG 20mg/kg (n = 18 rats)
Effectiveness of Nrf2 siRNA (n = 6 rats)
RT-qPCR, WB
mNSS score (n = 18 rats)
WB (n = 6 rats)
Brain water content (n = 6 rats)
FJC staining (n = 6 rats)
Total NF-κB p65,NLRP3,ASC,Caspase-1,IL-1β

D:

MRI and Hematoma Volume Evaluation

Experiment design and groups:

Thirty rats were used in this study. Rats were randomly divided into five groups, namely, sham group, ICH + vehicle-1 24 h, ICH + ILG 20 mg/kg 24 h, ICH + vehicle-1 72 h, ICH + ILG 20 mg/kg 72 h. At the corresponding time points, rats were performed magnetic resonance imaging (MRI) and then hematoma volume evaluations were carried out (n = 6).

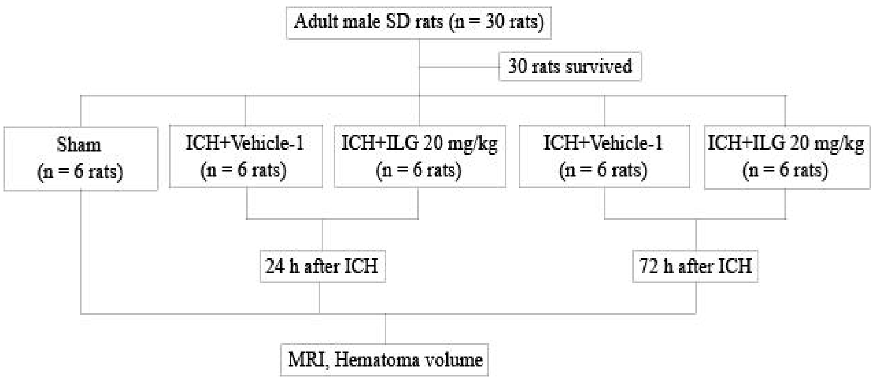

Methods:

MRI was conducted at 24 h and 72 h after ICH induction using a 7.0-T small-animal PharmaScan 70 / 16 MRI scanner (Bruker, USA) as previously performed[1, 2]. The rats were anesthetized with isoflurane and T2-weighted images (T2WI) were obtained using following parameters: Resolution matrix = 256 × 256, Field of view (FOV) = 35 mm × 35 mm, Slice number = 10, Slice thickness = 0.8 mm, slice gap = 0, TR / TE = 2500 ms / 33 ms. A skillful technician performed the image acquisition in a blinded manner[1, 2].

After MRI being performed, rats were deeply anesthetized and sacrificed to obtain whole brain, then serially sliced (2 mm thickness) anterior and posterior to the needle

entry site (identifiable on the brain surface). Coronal brain slices were photographed with a digital camera to get digital images. Then, the images were analyzed and hematoma volume was computed by using an Image J software package (National Institutes of Health, Baltimore, MD)[3, 4].

References:

[1]. Chen, Q., et al., Chronic hydrocephalus and perihematomal tissue injury developed in a rat model of intracerebral hemorrhage with ventricular extension. Transl Stroke Res, 2015. 6(2): p. 125-32.

[2]. MacLellan, C.L., et al., Intracerebral hemorrhage models in rat: comparing collagenase to blood infusion. J Cereb Blood Flow Metab, 2008. 28(3): p. 516-25.

[3]. Fang, H., et al., CD36-mediated hematoma absorption following intracerebral hemorrhage: negative regulation by TLR4 signaling. J Immunol, 2014. 192(12): p. 5984-92.

[4]. Liew, H.K., et al., Systemic administration of urocortin after intracerebral hemorrhage reduces neurological deficits and neuroinflammation in rats. J Neuroinflammation, 2012. 9: p. 13.

E:

Additional file 2

Effects of ILG on the hematoma volume and expansion at 24 h and 72 h after ICH (A, B) and effects of ILG on the number of $CD68^+$, $Iba\text{-}1^+$ cells in the perihematomal brain tissue at 24 h after ICH (C-E). Representative MRI T2WI images (A) and quantitative analyses of hematoma volume (B) (n = 6 rats / group). Representative microscopic images (C) and quantitative analyses of $CD68^+$, $Iba\text{-}1^+$ cells (D, E) (n = 6 rats / group). Scale bar = 20 μm. Values are reported as means ± SD. ** $p < 0.01$, * p

< 0.05.

Additional file 3

Mechanism diagram. Underlying molecular mechanisms of ILG’s neuroprotective effects on the early brain injury after ICH induction. ILG alleviated the early brain injury following ICH may be involved in the regulation of ROS and / or NF-κB on the activation of NLRP3 inflammasome pathway by the triggering of Nrf2 activity and the induction of Nrf2-mediated antioxidant system.
