# Supplementary figures and images for: Isoliquiritigenin alleviates early brain injury after experimental intracerebral hemorrhage via suppressing ROS- and/or NF-κB-mediated NLRP3 inflammasome activation by promoting Nrf2 antioxidant pathway
Source: J Neuroinflammation. 2017 Jun 13;14:119. doi: 10.1186/s12974-017-0895-5 (PMC5470182; doi:10.1186/s12974-017-0895-5)

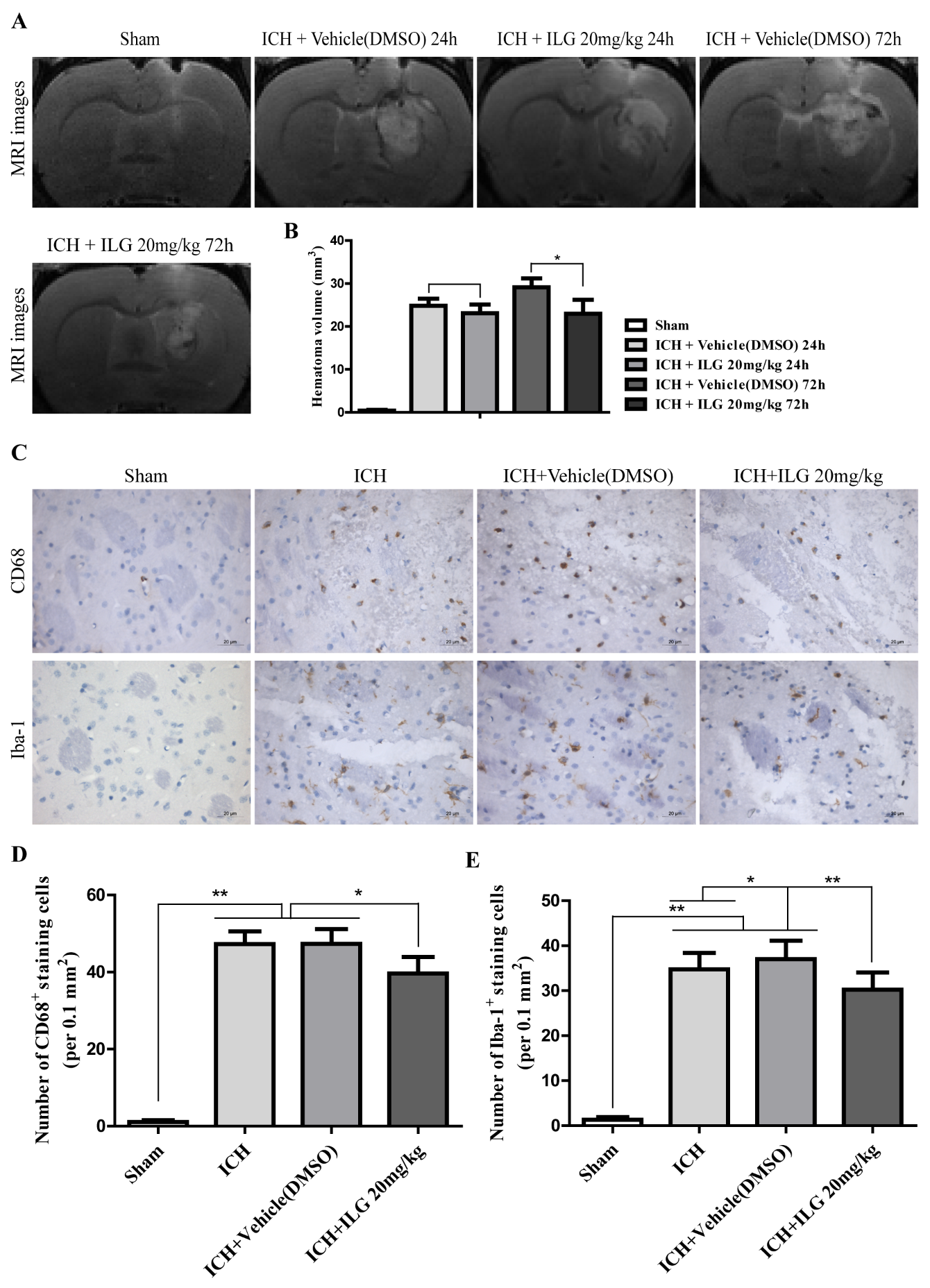

Supplement: Supplementary file 2 — Effects of ILG on the hematoma volume and expansion at 24 h and 72 h after ICH (a, b) and effects of ILG on the number of CD68+, Iba-1+ cells in the perihematomal brain tissue at 24 h after ICH (c-e). Representative MRI T2WI images (a) and quantitative analyses of hematoma volume (b) (n = 6 rats / group). Representative microscopic images (c) and quantitative analyses of CD68+, Iba-1+ cells (d, e) (n = 6 rats /group). Scale bar = 20 μm. Values are reported as means ± SD. ** p < 0.01, * p < 0.05. (TIF 7318 kb) [file 12974_2017_895_MOESM2_ESM.tif]

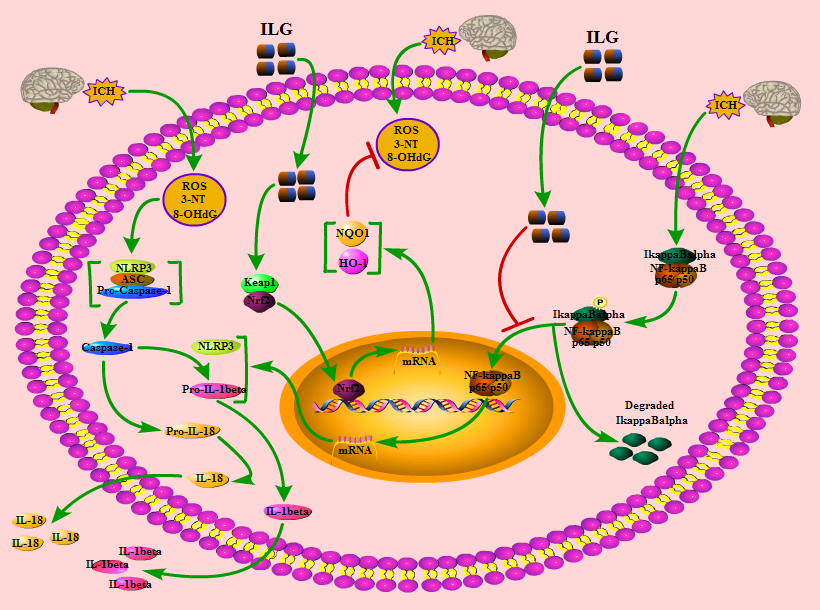

Supplement: Supplementary file 3 — Mechanism diagram. Underlying molecular mechanisms of ILG’s neuroprotective effects on the early brain injury after ICH induction. ILG alleviated the early brain injury following ICH may be involved in the regulation of ROS and / or NF-κB on the activation of NLRP3 inflammasome pathway by the triggering of Nrf2 activity and the induction of Nrf2-mediated antioxidant system. (TIF 232 kb) [file 12974_2017_895_MOESM3_ESM.tif]
